# A highly fluorinated functionalized magnetic covalent organic framework for high-efficient extraction of aflatoxins from diverse food matrices

**DOI:** 10.1016/j.fochx.2025.102863

**Published:** 2025-08-05

**Authors:** Dan Wei, Jianliang Li, Yixuan Ni, Qiao Deng, Ming Guo, Zuxin Wang, Huizhen Wu, Xu Wang, Jingjing Xu

**Affiliations:** a School of Laboratory Medicine and Bioengineering, Hangzhou Medical College, Hangzhou, Zhejiang 311300, China; b Key Laboratory of Biomarkers and In Vitro Diagnosis Translation of Zhejiang Province, Hangzhou 310058, China; c College of Biology and Environmental Engineering, Zhejiang Shuren University, Hangzhou 310015, China; d Zhejiang Chemical Production Quality Inspection Co., Ltd, Hangzhou 310023, China; e School of Economics and Management, Universiti Putra Malaysia, Kampong Serdang Belah, Selangor 43000, Malaysia

**Keywords:** Aflatoxins, Foods, Highly fluorinated functionalization, Magnetic covalent organic framework, Magnetic solid phase extraction

## Abstract

Aflatoxins, carcinogenic mycotoxins, pose a global threat to food safety. Rapid, sensitivity and reliable quantitative detection of trace aflatoxins in diverse food matrices is critical for safeguarding public health but remains challenging. To address this, rational design of adsorbents with abundant specific binding sites offers a promising solution. Herein, a novel fluorine-rich magnetic covalent organic framework (4F-COF@Fe_3_O_4_) was facilely synthesized, featuring a high specific surface area (945.438 m^2^ g^−1^). Computational and experimental investigations confirmed that synergistic interactions and large specific surface area were key to its enhanced extraction performance. Utilizing only 2 mg of adsorbent, 4F-COF@Fe_3_O_4_ based magnetic solid phase extraction coupled with HPLC-MS/MS was developed for analysis of aflatoxins in nine types of food matrices. This method exhibited good linearity (0.010–50 μg kg^−1^), low limits of detection (0.001–0.029 μg kg^−1^), and satisfactory recovery (71.5–112.8 %). The results demonstrated the potential of 4F-COF@Fe_3_O_4_ as a superior adsorbent for trace aflatoxins monitoring.

## Highlights

• **One-pot catalyzed Schiff base synthesis yields 4F-COF@Fe**_**3**_**O**_**4**_ **with high surface area and magnetism.**
• DFT and experiments reveal F-mediated adsorption sites for efficient AFs capture.
• High fluorination enables highly efficient aflatoxins extraction using minimal adsorbent.
• Sensitive AFs detection validated across nine diverse food matrices.

## 1 Introduction

Aflatoxins (AFs) are highly toxic and carcinogenic mycotoxins, primarily produced by *Aspergillus flavus* and *Aspergillus parasiticus* (Schamann, Soukup, Geisen, Kulling, & Schmidt-Heydt, 2024). These hazardous compounds can contaminate a wide range of foods, particularly cereals, fruits, edible oils, and milk, posing acute and chronic health risks, such as carcinogenicity, immunotoxicity, mutagenicity, hepatotoxicity, neurotoxicity, and genotoxicity (Kong et al., 2022). Among the AFs and their metabolites in global food chains, aflatoxins B1 (AFB1), B2 (AFB2), G1 (AFG1), G2 (AFG2), and M1 (AFM1) are the most prevalent, with AFB1 being the most potent and classified as a group I carcinogen by the International Agency for Research on Cancer (Mahmoud et al., 2025). AFs contamination typically occurs during crops growth, harvesting, storage, or processing, particularly in regions with high humidity and temperatures (Okechukwu, Adelusi, Kappo, Njobeh, & Mamo, 2024). In response to these growing risks, numerous countries and regions worldwide have established the strict limits for maximum permissible levels for total AFs or individual toxins in foods (Castell, Arroyo-Manzanares, Viñas, Lopez-García, & Campillo, 2024). Therefore, the rapid, sensitive and reliable analytical methods for AFs detection are critical for compliance with stringent regulatory limits and effectively safeguarding public health.

Current methods for AFs detection are broadly classified into three main categories: immunochemical assays, spectroscopic techniques, and chromatographic approaches (Niaz et al., 2025). While immunoassays and spectroscopic techniques are commonly used for screening, they often lack the specificity and sensitivity required for trace-level quantification (Levasseur-Garcia, 2018; Zhang & Banerjee, 2020). In comparison, chromatographic methods, particularly HPLC-MS/MS, have emerged as the dominant analytical technique due to the superior sensitivity, specificity, accuracy, and ability to analyze complex food matrices (Vargas Medina, Bassolli Borsatto, Maciel, & Lanças, 2021). An efficient and effective sample preparation remains indispensable to minimize matrix interferences and enrich AFs, thereby maximizing instrumental performance and ensuring reliable quantification (Niaz et al., 2025). Several traditional techniques, such as liquid-liquid extraction (LLE) and QuEChERS often suffer from excessive solvent consumption or insufficient selectivity (Azooz, Al-Wani, Gburi, & Al-Muhanna, 2022; Wang, Li, Wang, Wu, & Shi, 2024), and conventional solid-phase extraction (SPE) is often laborious and time-consuming (Azooz, Tuzen, Mortada, & Ullah, 2022). To address these challenges, magnetic solid-phase extraction (MSPE) has emerged as a powerful alternative. By incorporating magnetic nanoparticles as adsorbents, MSPE offers distinct advantages, such as simplified operations, reduced organic solvent consumption, rapid solid-liquid separation, and convenient adsorbent reusability enabled by an external magnetic field (Castell et al., 2024; Zhang et al., 2023). These features make MSPE well-suited for the rapid, selective, high-throughput extraction of trace AFs from complex food samples. Notably, since the magnetic adsorbent is central to the overall performance of the MSPE, the design and development of efficient magnetic materials, specifically tailored to the molecular characteristics of targets, are critically important (Fu et al., 2023).

To date, various magnetic adsorbents have been explored for AFs extraction, each with distinct advantages and limitations (Yu et al., 2025). For instance, immunomagnetic beads are widely employed due to their remarkable selectivity and specificity for AFs. However, their practical application is constrained by their high cost, complex synthesis, restricted reusability, and potential antibody denaturation when exposed to certain chemical solvents, and the risk of cross-reactivity and non-specific interactions (Singh & Mehta, 2020). In this context, covalent organic frameworks (COFs) have gained attention for their tunable porosity, high surface area, and chemical and thermal stability (Li, Lv, et al., 2022; Li, Xu, et al., 2022). Their large surface area introduces more active binding sites, making their potential for molecular recognition. Furthermore, their regular and tailored pore structure of COFs facilitates efficient mass-transfer, significantly enhancing rapid extraction of target molecules (Castell et al., 2024; Fu et al., 2023). Consequently, recent studies have explored COFs for the enrichment and detection of AFs and their structurally similar compounds like steroid ergometrine (ST) (Li, Qin, et al., 2023; Li, Yang, et al., 2023, Wei et al., 2023; Wei et al., 2025; Li, Lv, et al., 2022; Li, Xu, et al., 2022). Despite these achievements, reported magnetic COFs often face challenges in achieving optimal capture efficiency and selectivity for AFs, particularly when dealing with diverse and complex food matrices. Whereas COFs possess intrinsic structural advantages, the specificity of COFs is often insufficient because most primarily rely on the non-specific or relatively undifferentiated interactions for AFs capture, thus restricting the application in the presence of complicated interferences. Meanwhile, the incorporation of magnetic nanoparticles (e.g., Fe_3_O_4_) into COF frameworks enables rapid and facile magnetic separation, yet compromises surface area and porosity, leading to reduced binding site availability and impaired diffusion efficiency (Wang et al., 2024; Yang et al., 2022). Additionally, the synthesis of COFs typically requires high temperature (Fang et al., 2025) or involves multi-step procedures (Li, Yang, et al., 2023). Therefore, the rational design of a magnetic COF that simultaneously addresses these issues is urgently needed. In accordance with the characteristic difuran rings, coumarin structures, and oxygen-containing groups of AFs, which are rich in electron-rich regions and sites for potential polar interactions, it is hypothesized that introducing highly electronegative fluorine atoms into magnetic COFs would create specific F-mediated interaction sites for AFs. This hypothesis lies in synergistic interactions of F—O, F–π, F-induced van der Waals forces and H-bonding interactions to enable efficient AFs capture (Bi et al., 2024; Fang et al., 2025; Wang et al., 2024; Zang, Ren, He, Chen, & Hu, 2022). However, research on the facile synthesis of such fluorine-rich magnetic COFs for efficient extraction of AFs across diverse food matrices remains limited.

Herein, a novel fluorine-rich magnetic TAPT-COFs nanocomposite (4F-COF@Fe_3_O_4_) was successfully synthesized using a convenient one-pot approach from 2,4,6-tris(4-aminophenyl)-1,3,5-triazine (TAPT) and 2,3,5,6-tetrafluoroterephthalaldehyde (4F-PDA) under moderate conditions. The as-prepared 4F-COF@Fe_3_O_4_ was systematically characterized and subsequently employed as the adsorbent for efficient MSPE of AFB1, AFB2, AFG1, AFG2 and AFM1 from food matrices. Attributed to the synergistic interactions and high specific surface area, the 4F-COF@Fe_3_O_4_ demonstrated remarkably enhanced extraction capability towards the target AFs. The underlying extraction mechanism was comprehensively investigated through the material characterizations, adsorption experiments, and density functional theory calculations (DFT). Consequently, a rapid, sensitive and reliable 4F-COF@Fe_3_O_4_-based MSPE-HPLC-MS/MS method was successfully developed for the analysis of trace levels of AFs across nine food matrices.

## 2 Materials and methods

### 2.1 Materials

2,4,6-tris(4-aminophenyl)-1,3,5-triazine (TAPT), 2,3,5,6-tetrafluoroterephthalaldehyde (4F-PDA), *o*-dichlorobenzene, *n*-butanol, acetonitrile (ACN) were purchased by Aladdin (Shanghai, China). Ethanol, tetrahydrofuran, hexane, benzaldehyde, aniline, ferroferric oxide (Fe_3_O_4_), ferric nitrate (Fe(NO_3_)_3_), and acetic acid (HAc) were purchased by Macklin (Shanghai, China). Ultrapure water (UPW) was prepared with a Millipore Milli-Q system (Millipore, Bedford, MA, USA). Aflatoxin B1 (AFB1), aflatoxin B2 (AFB2), aflatoxin G1 (AFG1), aflatoxin G2 (AFG2), aflatoxin M1 (AFM1) standards (purity ≥99 %) were obtained from Sigma-Aldrich (St. Louis, MO, 141 USA). A series of working solutions with specific concentrations were prepared by stepwise dilution of a stock solution using the suitable solvent.

### 2.2 Apparatus

The morphological structure was obtained by scanning electron microscopy (SEM) (Sigma 300, ZEISS, Germany) and transmission electron microscopy (TEM) (Tecnai F20, FEI, USA). The crystal structure was performed on a ULTIMA IV X-ray diffraction (XRD) (Rigaku, Japan). The functional group structure was investigated on a Nicolet iS5 Fourier transform infrared spectrometer (FT-IR) (Thermo Fisher, USA). The pore structure was measured using N_2_ adsorption-desorption isotherms on a Micromeritics ASAP 2460 automatic volumetric instrument (Micromeritics, USA). The magnetic properties were measured using a Model 8604 Vibrating Sample Magnetometer (VSM) (Lakeshore, USA). X-ray photoelectron spectroscopy (XPS) investigation was performed on a Thermo Scientific K-Alpha (Thermo Fisher Scientific, USA) for material characterization.

Separation and detection of AFs was performed using an Agilent 1290 HPLC system coupled with a 6495 MS detector (Agilent, USA) and a Titank C18 column (3 mm × 150 mm, 3.0 μm, Phenomenex, China) maintained at 40 °C. The mobile phase consisted of 100 % ACN (phase A) and 5 mmol L^−1^ ammonium acetate aqueous solution (phase B), with a flow rate of 0.4 mL min^−1^. The gradient elution program was set as follows: 20 % A at 0 min, increased to 95 % A at 5 min, and returned to 20 % A at 6 min. The injection volume was 2 μL, and each sample was analyzed within 10 min. The mass spectrometer was operated in positive multiple reaction monitoring (MRM^+^) mode, with optimized conditions as follows: drying gas temperature at 300 °C, sheath gas temperature at 250 °C, drying gas flow rate at 5 L min^−1^, sheath gas flow rate at 10 L min^−1^, nebulizer pressure at 45 psi, capillary voltage at 3500 V, and nozzle voltage at 0 V. High-purity nitrogen was used as the drying, sheath, and nebulizer gas. Detailed MS parameters for AFs were listed in Table S1, and the corresponding HPLC chromatogram was shown in Fig. S1.

### 2.3 Synthesis of 4F-COF@Fe_3_O_4_

4F-COF@Fe_3_O_4_ composite was synthesized through schiff base condensation reaction, using Fe(NO_3_)_3_ as the catalyst and aniline as the inhibitor. Specifically, TAPT (72 mg), 4F-PDA (58 mg) were dissolved in a mixed solvent consisting of 20 mL *o*-dichlorobenzene and 15 mL *n*-butanol within a 150 mL three-necked flask. Then, Fe_3_O_4_ (150 mg), benzaldehyde (42 mL) and aniline (36 mL) were added to the solution, followed by sonication to ensure thorough dispersion. After that, Fe(NO_3_)_3_ (30 mg), *n*-butanol (5 mL) were slowly introduced, and the mixture was sonicated until complete dissolution. The resulting mixture was then maintained at 70 °C for 24 h. The obtained dark brown product was washed using tetrahydrofuran and alcohol, and dried under vacuum at 60 °C overnight.

### 2.4 Sample preparation

Samples of milk (whole fat liquid milk), cereals and cereal products (corn, rice, soybean, sweet potato, oats and bread), fruit (banana), edible oil (colleseed oil), were randomly collected from the local market in Hang Zhou, China. The obtained cereal, cereal product and fruit samples were homogenized. For each sample, 5 g for each homogenized corn, rice, soybean, sweet potato, oats, bread and banana sample, and 5 mL for each colleseed oil or milk sample were subjected to extraction. Then, the extraction of AFs from each sample was performed according to the previous reported studies with some modifications (Li, Yang, et al., 2023; Fang et al., 2025). Briefly, 10 mL of a solvent mixture of ACN and UPW (80/20, *v*/v) was added to to each sample. The mixture was vortexed for 1 min and sonicated for 30 min to ensure complete extraction. The resulting extract was evaporated to dryness under a nitrogen flow at 40 °C, reconstituted to 10 mL using water, and stored at −20 °C until use.

### 2.5 MSPE procedure

A total of 2 mg of 4F-COF@Fe_3_O_4_ was added to a 2 mL centrifuge tube containing 1 mL of either the standard solution or a prepared sample. The mixture was vortexed for 15 s to facilitate the adsorption of AFs onto the 4F-COF@Fe_3_O_4_ material. Following this, 4F-COF@Fe_3_O_4_ was magnetically separated, and 1 mL of a desorption solvent composed of ACN/UPW/HAc (90/9/1, v/v/v) was added. The mixture was vortexed for 3 min to release the adsorbed AFs. Then, the eluent was collected, filtered through a 0.22 μm membrane, and analyzed by HPLC-MS/MS.

### 2.6 Theoretical calculations

Quantum mechanical calculations were conducted using the ORCA quantum chemistry software (Version 6.0.0). The molecular structures of AFs, a periodic fragment of 4F-COF, and their corresponding complexes were modeled and optimized employing the r2SCAN-3c exchange-correlation functional, which combines the r2SCAN meta-GGA with D4 dispersion correction and geometrical counterpoise correction. The def2-mTZVPP basis set was selected for its suitability for light main-group elements. For rapid screening and prediction of optimal adsorption complexes, the GFN2-xTB method implemented in the xTB program was employed based on semiempirical quantum mechanical principles. To analyze and visualize potential interactions between AFs and 4F-COF, several approaches were applied, including the electrostatic potential (ESP) mapping, the independent gradient model based on the Hirshfeld partition (IGMH), and a series of singlet point energy calculations. ESP surface was analyzed to characterize its surface charge distribution and identify potential interaction sites. IGMH analysis was performed using Multiwfn 3.8 software, and the resulting IGMH isosurfaces were visualized using Visual Molecular Dynamics 1.9.3. Singlet point energy calculations were conducted on the optimized geometries using wB97M-V functional and ma-def2-TZVPP basis set. The interaction energy (*E*_*bind*_) was calculated using the following formula (Eq. (1)):

(1) $$E_{\mathrm{bind}}=E_{\mathrm{complex}}-\left(E_{A}+E_{B}\right)$$

where *E*_*complex*_ is the single point energy of the optimized complex structure, and *E*_*A*_ and *E*_*B*_ are the single point energies of the separately optimized individual components.

### 2.7 Statistical analysis

All experiments were conducted in triplicate, and the experimental results were recorded as the mean ± standard deviation (SD). Statistical significance was assessed using one-way analysis of variance (ANOVA) followed by Tukey's post-hoc test, performed with SPSS Statistics 26 software. A significance level of *p* < 0.05 is considered statistically significant. Most experimental data were plotted using Origin 2021 software.

## 3 Result and discussion

### 3.1 Design and preparation of 4F-COF@Fe_3_O_4_

To achieve rapid, selective extraction of AFs from complex food matrices with minimal adsorbent consumption, the design of a novel magnetic COFs adsorbent focuses on three key features: (a) abundant specific functional groups for target AFs interactions, (b) a high specific surface area and porosity to maximize binding site accessibility and promote mass transfer rate, and (c) magnetic properties for convenient separation (Castell et al., 2024).

Based on the structural characteristics of AFs, rich in electron-dense regions from oxygen-containing groups, aromatic structures, functionalized COF with highly electronegative fluorine atoms would introduce multiple specific interactions, such as F–O, F–π interactions (Bi et al., 2024; Fang et al., 2025; Wang et al., 2024) for effective AFs extraction. The fluorine-rich building monomer 4F-PDA was chosen to provide abundant fluorine-mediated interactions sites (e.g., H-bonding, electronegativity, dipole effects) essential for effective AFs adsorption. TAPT was selected for its triazine and aromatic amine groups, which enhances structural stability through triazine-aromatic conjugation and offers numerous π–π donors/acceptors within the framework. To facilitate easy separation and recycling, a magnetic Fe_3_O_4_ core was integrated. Furthermore, to overcome the limitations of conventional harsh or multi-step synthesis methods for magnetic COFs, a one-pot in-situ approach was developed to synthesize the 4F-COF@Fe_3_O_4_ composite on the Fe_3_O_4_ core under mild conditions.

**Fig. 1:**
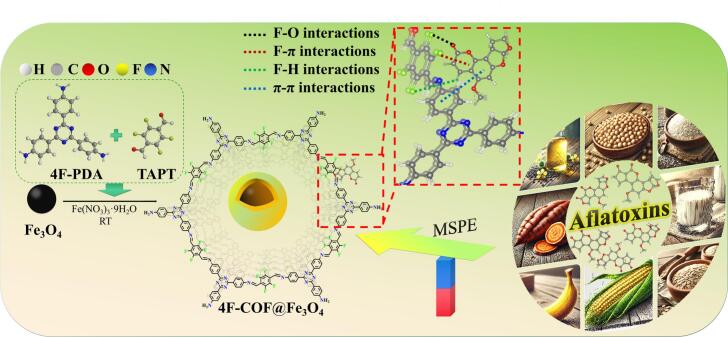
Schematic diagram of the adsorbent preparation and main extraction mechanism.

**Fig. 2:**
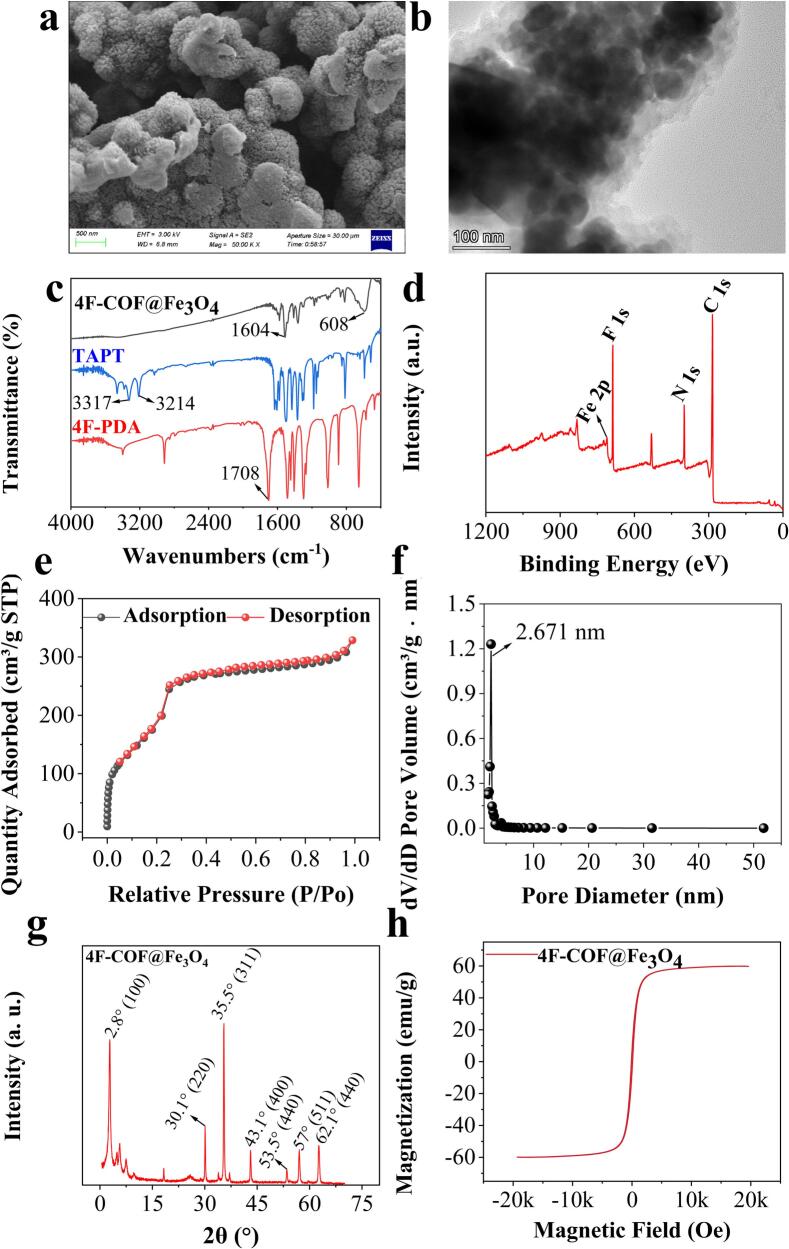
(a) SEM image (b) TEM of 4F-COF@Fe_3_O_4_, (c) FT-IR spectra of TAPT, 4F-PDA and 4F-COF@Fe_3_O_4_, (d) XPS spectra, (e) N_2_ adsorption-desorption analysis, (f) pore size, (g) XRD pattern, and (h) magnetization curve of the 4F-COF@Fe_3_O_4_.

Characterization confirmed the successful synthesis of the 4F-COF@Fe_3_O_4_ composite, exhibiting a well-defined crystalline structure, high surface are and porosity, strong magnetic responsiveness (Fig. 2a–h). These features collectively contribute to the superior extraction efficiency, excellent stability and good reusability of 4F-COFs@Fe_3_O_4_. Subsequently, 4F-COFs@Fe_3_O_4_ was used for the rapid and sensitive detection of AFs in various food samples, followed by HPLC-MS/MS analysis. Schematic diagram illustrating the adsorbent preparation and main extraction mechanism were presented in Fig. 1.

### 3.2 Characterization of 4F-COF@Fe_3_O_4_

The morphology of 4F-COF@Fe_3_O_4_ was characterized by SEM and TEM. As shown in Fig. 2a and b, the 4F-COF@Fe_3_O_4_ was nearly spherical with a rough and uneven surface and an average diameter of 35 nm. TEM imaging (Fig. 2b) further revealed a core-shell structure, where a COF layer with a thickness of about 15 nm successfully encapsulated the Fe_3_O_4_ core.

The FTIR spectra of 4F-COF@Fe_3_O_4_, 4F-PDA, and TAPT are presented in Fig. 2c. Characteristic stretching vibrations for the N—H bonds in TAPT (3214 cm^−1^, 3317 cm^−1^) and the C=O bond in 4F-PDA (1708 cm^−1^) disappeared in the spectrum of 4F-COF@Fe_3_O_4_. Instead, a new peak was observed at 1604 cm^−1^, corresponding to the the —C=N—. Additionally, the peak at 608 cm^−1^ was attributed to the Fe—O vibration of Fe_3_O_4_. These spectral changes indicate the successful condensation reaction between the monomers and the presence of the Fe_3_O_4_ core within composite. The XPS spectra provided additional provided further elemental and chemical state analysis of 4F-COF@Fe_3_O_4_. The XPS spectrum (Fig. 2d) confirmed the presence of F 1s peak, C 1s peak, N 1s peak, Fe 2p peak at 687.50 eV, 284.42 eV, 398.57 eV, and 710.56 eV, respectively. The deconvolution of C 1s peak revealed four distinct peaks attributed to C=C (284.19 eV), F—C (285.58 eV), C—C (287.27 eV), and C=N (290.39 eV), further supporting the successful synthesis of the fluorine-functionalized COF structure.

The surface area and porosity of 4F-COF@Fe_3_O_4_ were characterized by an N_2_ adsorption-desorption curve (Fig. 2e). The analysis Brunauer-Emmett-Teller (BET) surface area of 945.438 m^2^ g^−1^, an average pore size of 2.671 nm, an average pore volume of 0.570 cm^3^ g^−1^ (Fig. 2f). The nitrogen adsorption-desorption isotherm exhibited exhibited a type IV curve with mesoporous features. The high surface area and porosity are crucial for providing abundant adsorption sites and facilitating rapid mass transfer, thereby contributing to its efficient extraction performance for AFs.

The crystalline structure of 4F-COF@Fe_3_O_4_ was investigated by XRD. As shown in Fig. 2g, diffraction peaks were observed at 30.1°, 35.5°, 43.1°, 53.5°, 57°, and 62.1°, corresponding to the (1 0 0), (2 2 0), (3 1 1), (4 0 0), (4 4 0), and (5 1 1) planes, respectively, consistent with the crystallization of Fe_3_O_4_ (Hu et al., 2023; Wei et al., 2023). Furthermore, a sharp characteristic diffraction peak appeared at 2.8°, similar to the previous reported F-COF (Wang et al., 2024). All the results confirmed the successful formation of the ordered crystalline COF framework.

The magnetic properties of 4F-COF@Fe_3_O_4_ were determined by VSM experiment. The saturation magnetization value of 59.8 emu g^−1^ (Fig. 2h) is sufficient to ensure efficient and rapid magnetic separation of 4F-COF@Fe_3_O_4_ from complex sample matrices using an external magnetic field.

### 3.3 Optimization of MSPE conditions

#### 3.3.1 Magnetic adsorbent dosage

**Fig. 3:**
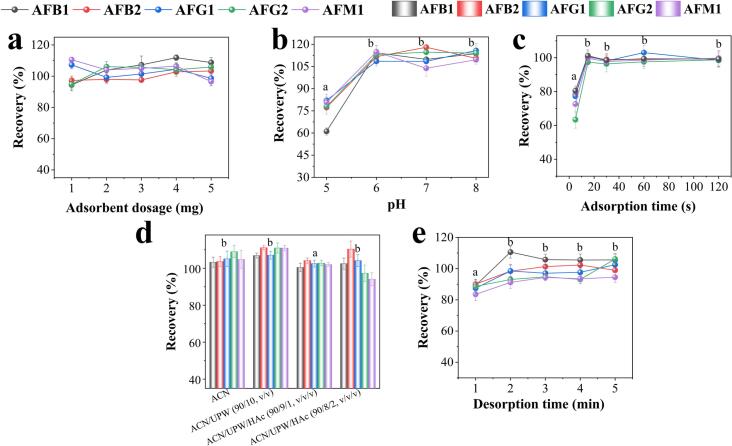
Optimization of the MSPE conditions: (a) adsorbent dosage, (b) sample pH, (c) adsorption time, (d) type of eluent, and (e) desorption time. Different lowercase letters (a, b) indicate a statistically significant difference (*p* < 0.05) according to Tukey post-hoc test, while the absence of letters indicate no significant difference (*p* > 0.05).

The dosage of magnetic adsorbent is a critical factor directly affecting the available active sites for targets adsorption, and thus determining the overall MSPE efficiency (Lin et al., 2020). In this study, the influence of adsorbent dosage was investigated in the range of 1–5 mg. As shown in Fig. 3a, the recoveries increased as adsorbent dosage increased from 1 to 2 mg, but plateaued with further increases from 2 to 5 mg. Single-factor ANOVA results confirmed that there was no significant difference in extraction performance for adsorbent dosages from 1 to 5 mg (*F* = 0.91, *p* > 0.05). The initial slight increase is attributed to the greater availability of adsorption sites with increasing adsorbent dosage from 1 to 2 mg. Beyond 2 mg, the available adsorption sites were sufficient to capture the target AFs present in the sample solution, effectively reaching an adsorption equilibrium. Therefore, to ensure efficient extraction while minimizing material consumption, 2 mg of 4F-COF@Fe_3_O_4_ was selected as the optimal dosage for subsequent MSPE experiments.

#### 3.3.2 Sample pH

The sample pH influences the extraction performance by affecting the chemical state of analytes and the surface properties of the adsorbent (Li, Lv, et al., 2022; Li, Xu, et al., 2022). The influence of sample pH on MSPE efficiency was investigated in the range of 5–8 adjusted by ammonia aqueous and HAc solution. As shown in Fig. 3b, the satisfactory recoveries (89.9 %–119.5 %) were observed within the pH range of 6–8. Statistical analysis indicated that the effect of pH was significant (*F* = 6.47, *p* < 0.05), with Tukey's post-hoc test revealing no significant difference among pH 6, 7, and 8, but a significant difference compared to pH 5. This suggests that within pH range of 6–8, the neutral or near-neutral state of AFs aligned well with the surface properties of the material, favoring Van der Waals force, π–π interactions and H-bonding interactions with the fluorinated COFs. Consequently, the pH range of 6–8 was selected for subsequent experiments.

#### 3.3.3 Adsorption time

Adsorption time is an important parameter affecting MSPE efficiency and serves as an indicator for adsorbent performance (Man et al., 2025). To achieve high MSPE efficiency, the adsorption time ranging from 5 to 120 s was evaluated. As shown in Fig. 3c, the extraction efficiency increased sharply within the first 15 s and plateaued thereafter. The ANOVA results showed that adsorption time had a highly significant effect on the extraction recovery (*F* = 59.07, *p* < 0.001), with Tukey's post-hoc analysis confirming that the recovery at 5 s was significantly lower than those at 15–120 s. The rapid achievement of high recovery (>95.8 %) with only 15 s can be ascribed to its favorable structural characteristics: the large specific surface area provides a high density of available adsorption sites, while the well-developed mesoporosity facilitates rapid mass transfer of the AFs from the solution to the surfaces of the material. The efficient mass transfer ensures quick access to the binding sites, thus leading to rapid achievement of adsorption equilibrium. As a result, 15 s was chosen as the optimal absorption time for achieving rapid and efficient extraction.

#### 3.3.4 Eluent solvent

Selecting an appropriate eluent is key to achieving efficient MSPE. According to previous studies (Niaz et al., 2025; Liu et al., 2022; Li, Yang, et al., 2023), the eluent solvents for AFs composed of organic solvents (ACN, methanol, ethanol, ethyl acetate, and dichloromethane) or mixtures with acid are typically used. Acidic ACN is often preferred due to its polarity and effectiveness in disrupting H-bonding interactions between the adsorbates (serving as H-bonding donors) and the adsorbent (acting as H-bonding acceptors). This facilitates efficient desorption of AFs from the adsorbent based on the “Like-Dissolves-Like” principle and hydrogen-bonding effects (Wang et al., 2024). Subsequently, several potential ACN-based eluents were investigated in terms of extraction recoveries, including ACN, ACN/UPW (90/10, *v*/v), ACN/UPW/HAc (90/9/1, v/v/v), ACN/UPW/HAc (90/8/2, v/v/v). As shown in Fig. 3d, all tested eluent solvents achieved extraction recoveries above 91.14 %. A significant difference was observed among the eluents (*F* = 7.82, *p* < 0.05). However, the ACN/UPW/HAc mixture (90/9/1, v/v/v) exhibited superior desorption capability and the lowest variability, which was statistically significant compared to other tested eluents according to Tukey's test. Consequently, ACN/UPW/HAc mixture (90/9/1, v/v/v) was chosen as the optimal eluent for following experiments.

#### 3.3.5 Desorption time

Desorption time is also a crucial factor for overall MSPE efficiency. Sufficient desorption time ensures the complete removal of the targets from the magnetic adsorbent (Yu et al., 2022). The effect of desorption time on MSPE recovery was then investigated, with desorption times ranging from 1 to 5 min. As shown in Fig. 3e, MSPE recovery increased with desorption time up to 3 min, then remained stable beyond 3 min. The effect of desorption time was statistically significant (*F* = 7.85, *p* < 0.05), with post-hoc tests indicating that the recovery at 1 min was significantly lower than those achieved at 2–5 min. This suggested that 3 min was sufficient for complete desorption of the adsorbed AFs from the 4F-COF@Fe_3_O_4_ into the eluent solution, effectively reaching desorption equilibrium. Consequently, a desorption time of 3 min was selected for further experiments.

In summary, the optimal parameters for 4F-COF@Fe_3_O_4_-based MSPE method were identified as follows: adsorbent dosage of 2 mg, sample pH in the range of 6–8, adsorption time of 15 s, eluent solvents of ACN/UPW/HAc (90/9/1, v/v/v), and desorption time of 3 min.

### 3.4 Reusability, stability and reproducibility of 4F-COF@Fe_3_O_4_

Reusability, stability and reproducibility are also crucial indicators of an ideal MSPE adsorbent. The reusability was evaluated by assessing the extraction recoveries over several MSPE cycles using AFs spiked sample solutions. As shown in Fig. S2, the recoveries of the target AFs remained above 84.2 % after ten consecutive cycles. The observations confirmed that 4F-COF@Fe_3_O_4_ can maintain good reusability, withstanding at least 10 consecutive MSPE cycles.

To further evaluate the structural stability, FTIR and XRD analyses of 4F-COF@Fe_3_O_4_ were conducted after immersion in acidic (pH 2), basic (pH 12) solutions and organic solvent (ethanol). FTIR spectra (Fig. S3) indicated that the Fe—O bonds (603 cm^−1^) in the Fe_3_O_4_ core and the C=N bonds (1602 cm^−1^) in the 4F-COF framework remained stable under acidic conditions (pH 2) and in organic solvents (ethanol). However, a slight decrease in peak intensity or peak broadening were observed under basic conditions (pH 12), indicating partial degradation. XRD patterns (Fig. S4) showed no significant changes in the distinct diffraction peaks under acidic or organic solvent conditions, whereas a noticeable decrease was observed in basic solutions, suggesting that the crystallinity of 4F-COF@Fe_3_O_4_ is well-preserved in acidic and organic environments, while moderately compromised under basic environment.

Reproducibility of 4F-COF@Fe_3_O_4_ was assessed by comparing the MSPE performance of pristine 4F-COF@Fe_3_O_4_ with that after exposure to acidic (pH 2), basic (pH 12) solutions, organic solvent (ethanol), and after storage for six months. As illustrated in Fig. S5, no significant differences in MSPE recoveries was observed, with recoveries maintaining above 86.1 %. Overall, the material retained its structural integrity and extraction capacity under acidic, basic and organic solvent conditions, and after 6 months storage, confirming the good stability, reusability, and reproducibility. Collectively, these characteristics make 4F-COF@Fe_3_O_4_ a promising material for MSPE of AFs.

### 3.5 Adsorption kinetics and isotherm

To assess the adsorption properties of 4F-COF@Fe_3_O_4_, the adsorption isotherm and adsorption kinetics experiments were conducted, utilizing AFM1 as a model molecule.

For adsorption isotherm experiments, 1 mg of 4F-COF@Fe_3_O_4_ was dispersed into 10 mL AFM1 solutions with initial concentrations ranging from 5 to 200 μg L^−1^. The mixtures were rigorously vortexed at 3000 rpm for 20 min. Details of the adsorption capacity calculation, as well as the Langmuir and Freundlich models, are provided in the Supplementary materials. Based on the adsorption curves of AFM1 on 4F-COF@Fe_3_O_4_ (Fig. S6), the adsorption capacity increased rapidly with the rise in its initial concentration. Moreover, the maximum adsorption capacity of 4F-COF@Fe_3_O_4_ was determined to be 185.16 μg g^−1^. The experimental adsorption data were analyzed using the Langmuir and Freundlich models. Notably, the Langmuir model displayed superior correlation (*R*_1_^2^ = 0.9836) in comparison to the Freundlich model (*R*_2_^2^ = 0.9744) (Fig. S6 and Table S2). The findings indicated that the adsorption of AFM1 onto 4F-COF@Fe_3_O_4_ mainly proceeded through a monolayer adsorption process.

For the adsorption kinetics experiments, 1 mg of 4F-COF@Fe_3_O_4_ was added to 10 mL of AFM1 solution at a concentration of 5 μg L^−1^. The mixture was vortexed at 2000 rpm for varying adsorption times ranging from 1 to 10 min. Details of the pseudo-first-order and pseudo-second-order kinetic models were provided in the Supplementary Materials. As shown in Fig. S7 and Table S3, the pseudo-second-order model (*R*_1_^2^ = 0.9694) provided a significantly better fit to the adsorption data compared to the pseudo-first-order model (*R*_2_^2^ = 0.3198). The experimental maximum adsorption capacity of AFM1 on 4F-COF@Fe_3_O_4_ was consistent with the predictions of the pseudo-second-order model. These kinetic results suggested the predominant role of chemical adsorption in the overall adsorption process.

### 3.6 Possible extraction mechanism

To elucidate the extraction mechanism, a comprehensive investigation combining experimental investigations, characterizations, and DFT simulations was conducted.

The average pore diameter of 4F-COF@Fe_3_O_4_ (2.67 nm, determined by BET analysis in Fig. 2f) exceeded the dimensions of AF molecules (0.81 × 0.81 × 1.13 nm). The size disparity facilitates the efficient diffusion and mass transfer of AF molecules within the pore channels, contributing to enhanced extraction performance.

**Fig. 4:**
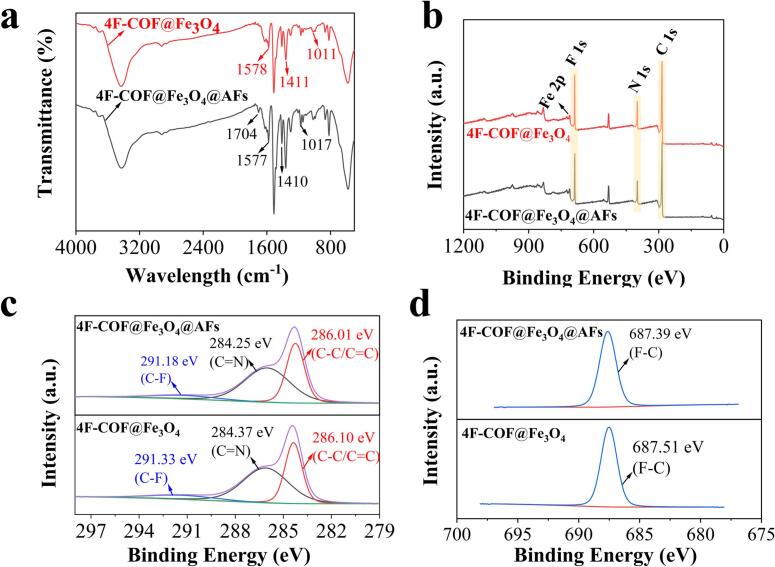
(a) FT-IR spectra, (b) XPS full-scan spectra, (c) C1s spectra and (d) F1s spectra of 4F-COF@Fe_3_O_4_ before (4F-COF@Fe_3_O_4_) and after AFs adsorption (4F-COF@Fe_3_O_4_@AFs).

FTIR and XPS analyses were performed to reveal the extraction mechanism by comparing the spectra of 4F-COF@Fe_3_O_4_ before and after AFs adsorption (4F-COF@Fe_3_O_4_@AFs). In the FT-IR spectra (Fig. 4a), the C=O stretching vibration peak of 1704 cm^−1^ observed in 4F-COF@Fe_3_O_4_@AFs proved the successful adsorption of AFs. Shift in the stretching vibration of C—C and C=C from 1411 cm^−1^, 1578 cm^−1^ to 1410 cm^−1^, 1577 cm^−1^ in 4F-COF@Fe_3_O_4_@AFs, respectively, suggested the formation of π–π interactions between the adsorbent and the AF molecules. A shift in the stretching vibration peak from 1011 cm^−1^ to 1017 cm^−1^ in 4F-COF@Fe_3_O_4_@AFs was also observed (Fig. 4a), which was ascribed to the formation of the F—O, F—H and F–π bonding interactions. These interactions were further confirmed through the XPS analysis (Fig. 4b-d). As depicted in Fig. 4c, the C 1s spectrum of 4F-COF@Fe_3_O_4_ was deconvoluted into peaks corresponding to C—F (291.33 eV), C=N (284.37 eV) and C-C/C=C (260.10 eV). The reduction in binding energies of C—C/C=C (286.01 eV), C=N (284.25 eV) and C—F (291.18 eV) observed in 4F-COF@Fe_3_O_4_@AFs further confirmed the occurrence of π–π stacking interactions and F—O, F—H and F–π bonding interactions. The shift in the binding energy of F 1s (decreasing from 687.51 eV to 687.39 eV) on 4F-COF@Fe_3_O_4_ after AFs adsorption also validated the presence of the fluorine-based interactions (Fig. 4d).

**Fig. 5:**
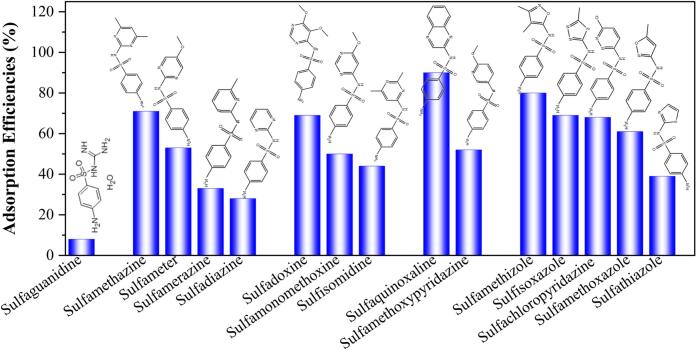
4F-COF@Fe_3_O_4_ adsorption efficiencies for sulfonamides.

Sulfonamide adsorption experiments using 4F-COF@Fe_3_O_4_ were conducted to investigate the potential roles of oxygen-containing groups and aromatic structures of the adsorbates in adsorption efficiencies. As illustrated in Fig. 5, 4F-COF@Fe_3_O_4_ exhibited efficient adsorption efficiencies for four types of sulfonamides, such as sulfaquinoxaline (90.3 %), sulfamethizole (80.6 %), sulfamethazine (71.2 %) and sulfisoxazole (68.9 %). Comparisons of similarly structured sulfonamides revealed that trends in extraction efficiency correlated with structural features. For instance, among sulfamethazine, sulfameter, sulfamerazine, and sulfadiazine, the extraction efficiencies followed the descending order: sulfamethazine (71.2 %) > sulfameter (52.8 %) > sulfamerazine (33.1 %) > sulfadiazine (27.9 %). Similarly, for sulfadoxine, sulfamonomethoxine, and sulfisomidine, the extraction efficiencies decreased as follows: sulfadoxine (69.1 %) > sulfamonomethoxine (52.8 %) > sulfisomidine (43.9 %). These trends can be attributed to the larger aromatic structures and extended conjugation present in certain sulfonamide molecules (e.g., sulfaquinoxaline, sulfamethazine), which facilitate π–π stacking interactions with 4F-COF@Fe_3_O_4_. Furthermore, the presence of electron-donors groups like methoxy, methyl (e.g., sulfamethizole, sulfisoxazole) could enhance polar interactions, such as F—O bonding, F–π bonding interactions and electrostatic interactions with 4F-COF@Fe_3_O_4_. In contrast, compounds (e.g., sulfaguanidine) with limited aromatic structures or oxygen groups exhibited notably lower adsorption rates. These finding highlighted the significant role of aromatic structures and oxygen-containing groups, as previously indicated for achieving efficient extraction of AFs by 4F-COF@Fe_3_O_4_.

**Fig. 6:**
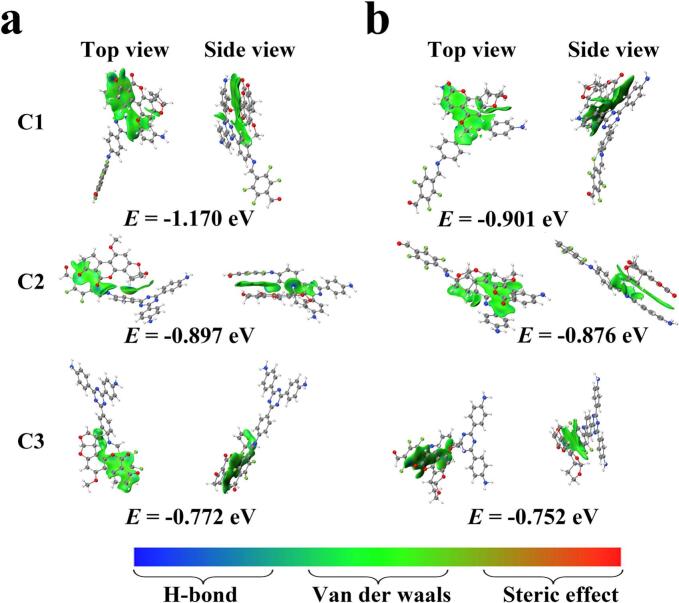
IGMH maps of the complexes of 4F-COF and (a) AFB1, (b) AF skeleton.

**Fig. 7:**
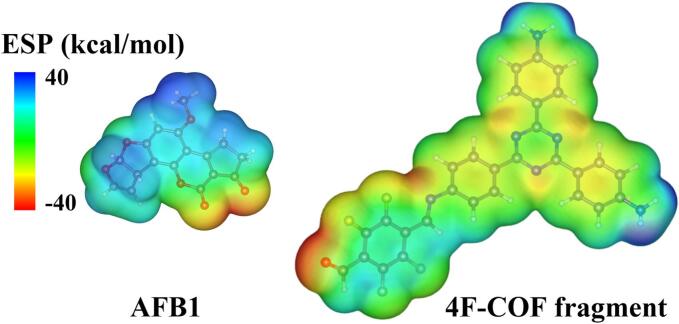
ESP maps of AFB1 and the fragment of 4F-COF@Fe_3_O_4_.

To further elucidate the extraction mechanism, DFT calculations involving singlet point energy, IGMH, and ESP mappings were systematically performed. AFB1 and a hypothetical AF skeleton molecule (representing the fundamental structure of AFs) served as as model compounds. Initial screening for optimal adsorption complexes (C1, C2, C3) were performed using the GFN2-xTB method. IGMH analysis provided visual and quantitative insights into the nature and intensity of the interactions within the optimal complexes. As shown in Fig. 6, the green regions on the gradient isosurfaces of complexes C1, C2 and C3 indicated that van der Waals forces and π–π stacking interactions predominantly governed the weak interactions between the 4F-COF and the AF model molecules. The predominance was likely driven by the strong dipole moments of the C—F bonds in 4F-COF and the C=O/C—O bonds in AFs, which promote van der Waals interactions via dipole-dipole interactions, dipole-induced dipole interactions, and dispersion forces. Furthermore, the high electronegativity of fluorine further influences the electron distribution of aromatic rings and double bonds in 4F-COF, indirectly enhancing the van der Waals contributions. Singlet point energy calculations (Fig. 6) yielded negative adsorption energies (*E*_*ads*_) for all complexes, indicating thermodynamically favorable adsorption. Among the adsorption models, the complex C1 adsorption models exhibited the most negative *E*_*ads*_, suggesting stronger π–π stacking interactions. ESP maps (Fig. 7) revealed complementary electrostatic interactions. The electron-rich regions of AFB1 (e.g., oxygen-containing groups) electrostatically attracted the positively charged areas of 4F-COF. While the electropositive regions of AFB1 (e.g., hydrogen atoms) aligned with the highly electronegative region of fluorine atoms in 4F-COF. These findings confirmed the significant role of electrostatic interactions in overall extraction process. All the DFT calculations consistently demonstrated that the extraction mechanism of AFs onto 4F-COF@Fe_3_O_4_ involved synergetic interactions of van der Waals forces, π–π stacking, and electrostatic interactions.

Overall, 4F-COF@Fe_3_O_4_, rich in aromatic and fluorine groups, offers abundant adsorption sites, facilitates rapid mass transfer, and enables specific interactions. Among the synergistic effects, π–π stacking and fluorine-driven van der Waals forces played a dominant role, complemented by the hydrogen bonding, electrostatic interactions and favorable pore-matching effects. Collectively, these interactions contribute to the rapid, selective, and efficient extraction of AFs from complex food matrices. The primary extraction mechanism of AFs by 4F-COF@Fe_3_O_4_ is illustrated in Fig. 1.

### 3.7 Selectivity test

To assess the selectivity of 4F-COF@Fe_3_O_4_, its extraction performance was examined across various frequently detected mycotoxins, including OTA, OTB, T-2, and STG, under optimal MSPE conditions. The results demonstrated that 4F-COF@Fe_3_O_4_ achieved superior extraction efficiencies for AFs, ranging from 93.8 % to 106.9 %, compared to other mycotoxins such as OTA (71.2 %), OTB (62.8 %), STG (63.7 %), T-2 (51 %), ZEN (70.1 %), α-ZEL (65.1 %) and β-ZEL (60.8 %). This selectivity can be attributed to the multiple interactions, such as the strong π–π interactions with aromatic-rich structures in AFs, van der Waals forces, hydrogen bonding and electrostatic interactions with polar oxygen-containing groups in AFs, and the pore matching effects of the 4F-COF@Fe_3_O_4_. Furthermore, the optimized MSPE parameters, combined with specific retention times, and MS characteristics of AFs, highlight the potential of the developed 4F-COF@Fe_3_O_4_-based MSPE HPLC-MS/MS method for the rapid, selective and reliable detection of AFs in complex food matrices.

### 3.8 Matrix effect

Co-extracted components in complex food samples often interfere with the AFs analysis, impacting the accuracy of the developed method. Matrix effects (ME, %) serve as an important indicator of the anti-matrix interference ability of the method. The ME was investigated by comparing the slope of matrix matched calibration curve with that of solvent calibration curve. The ME was calculated using the following formula (Eq. (2)):

(2) $$\mathrm{ME}\;\left(\%\right)=\left[\left(K_{2}-K_{1}\right)/K_{1}\right]\times 100$$

where *K*_*1*_ is the slope of the solvent calibration curve and *K*_*2*_ is the slope of matrix matched calibration curve. The ME value within ±20 % was considered to be ignored (Jiang, Cheng, Du, & Wang, 2023). As listed in Table 1, no significant ME was observed in soybean, sweet potato, rice, bread, colleseed oil, banana and whole fat liquid milk (−19.72 %–15.78 %), except for oat (a slight signal inhibition for AFB_1_, AFB_2_, AFG_1_, and AFG_2_), and corn (the strong signal enhancement for AFB_2_ and AFG_2_, and an inhibition of AFB_1_ and AFG_1_). To eliminate the MEs, the matrix-matched calibration curves were established at six different concentration levels (Zhao, Liu, Zhang, Zhou, & Yang, 2021).

**Table 1:** Analytical results of real samples using the developed method.

Matrices	Analytes	Linearity (μg kg^−1^)	Linear equation	*R*^2^	LOD (μg kg^−1^)	LOQ (μg kg^−1^)	ME (%)	RSD_*r*_ (%, *n* = 3)	RSD_*R*_ (%, *n* = 6)
Soybean	AFB1	0.010–50	y = 20,959x − 706	0.9991	0.002	0.010	−3.85	6.3	7.9
	AFB2	0.040–50	y = 4710x − 832	0.9991	0.009	0.040	−0.59	5.8	5.9
	AFG1	0.070–50	y = 7900x − 251	0.9996	0.019	0.070	2.51	4.9	9.1
	AFG2	0.100–50	y = 1563x − 278	0.9994	0.029	0.100	−1.79	5.7	7.9
	AFM1	0.050–50	y = 4529x + 8	0.9997	0.014	0.050	3.20	6.6	7.1
Banana	AFB1	0.010–50	y = 22,533x − 12,426	0.9988	0.003	0.010	3.37	4.8	4.2
	AFB2	0.060–50	y = 4869x − 1952	0.9989	0.017	0.060	2.76	5.1	6.8
	AFG1	0.030–50	y = 7995x + 2457	0.9894	0.007	0.030	3.74	4.9	5.1
	AFG2	0.100–50	y = 1592x + 449	0.9892	0.026	0.100	0.03	5.5	7.1
	AFM1	0.020–50	y = 4869x + 580	0.9935	0.005	0.020	10.94	3.8	5.0
Whole fat liquid milk	AFB1	0.010–50	y = 17,915x − 3776	0.9996	0.001	0.010	−17.82	6.5	7.1
	AFB2	0.050–50	y = 3998x − 1937	0.9990	0.014	0.050	−15.62	7.1	6.9
	AFG1	0.080–50	y = 6425x − 1084	0.9988	0.024	0.080	−16.63	5.9	7.1
	AFG2	0.050–50	y = 1303x − 23	0.9964	0.014	0.050	−18.11	6.3	8.5
	AFM1	0.025–50	y = 4886x − 3317	0.9985	0.006	0.025	11.32	5.5	5.8
Sweet potato	AFB1	0.020–50	y = 24,438x − 11,754	0.9999	0.005	0.020	−18.66	4.8	4.6
	AFB2	0.020–50	y = 5320x − 1343	0.9999	0.005	0.020	−19.72	6.2	7.7
	AFG1	0.050–50	y = 7642x − 1517	0.9998	0.014	0.050	−19.35	7.1	6.9
	AFG2	0.075–50	y = 1729x − 536	0.9998	0.019	0.075	−19.31	7.8	7.9
	AFM1	0.010–50	y = 5940x − 242	0.9998	0.001	0.010	7.69	9.4	10.5
Oat	AFB1	0.010–50	y = 22,487x − 3741	0.9996	0.001	0.010	−25.15	5.6	7.1
	AFB2	0.10–50	y = 4918x − 417	0.9992	0.025	0.100	−25.80	7.4	10.1
	AFG1	0.10–50	y = 7437x − 898	0.9997	0.027	0.100	−21.52	6.1	8.2
	AFG2	0.050–50	y = 1609x + 34	0.9986	0.010	0.050	−24.89	5.4	6.9
	AFM1	0.040–50	y = 5639x + 308	0.9986	0.012	0.040	2.22	6.8	8.2
Colleseed oil	AFB1	0.050–50	y = 14,087x − 14,612	0.9995	0.008	0.050	15.53	3.8	3.8
	AFB2	0.050–50	y = 9308x − 12,638	0.9990	0.012	0.050	9.87	4.7	8.8
	AFG1	0.10–50	y = 5266x − 5290	0.9992	0.017	0.100	10.30	4.2	4.1
	AFG2	0.075–50	y = 3403x − 2604	0.9975	0.016	0.075	0.99	5.3	7.9
	AFM1	0.025–50	y = 5078x − 1583	0.9960	0.006	0.025	3.96	6.1	5.9
Rice	AFB1	0.050–50	y = 14,538x − 15,228	0.9987	0.008	0.050	15.10	3.9	5.0
	AFB2	0.050–50	y = 9523x − 10,723	0.9981	0.013	0.050	12.41	4.8	6.1
	AFG1	0.025–50	y = 5281x − 2913	0.9989	0.006	0.025	10.62	5.5	8.2
	AFG2	0.050–50	y = 3619x − 3444	0.9936	0.012	0.050	7.38	4.1	6.3
	AFM1	0.060–50	y = 5440x − 5945	0.9974	0.017	0.060	11.37	5.7	7.2
Corn	AFB1	0.010–50	y = 21,942x − 4235	0.9998	0.001	0.010	−26.96	1.3	5.1
	AFB2	0.050–50	y = 10,012x − 1117	0.9998	0.012	0.050	51.08	8.0	10.0
	AFG1	0.075–50	y = 7545x − 2862	0.9997	0.022	0.075	−20.38	2.7	7.7
	AFG2	0.020–50	y = 3551x − 1505	0.9998	0.004	0.020	65.75	3.0	6.5
	AFM1	0.020–50	y = 5505x − 957	0.9997	0.005	0.020	−0.21	2.2	4.9
Bread	AFB1	0.020–50	y = 18,902x − 6590	0.9915	0.005	0.020	9.86	3.5	3.3
	AFB2	0.050–50	y = 10,945x − 11,909	0.9965	0.015	0.050	−12.01	4.9	5.1
	AFG1	0.150–50	y = 8069x − 1908	0.9990	0.045	0.150	−15.78	6.9	7.0
	AFG2	0.10–50	y = 3010x − 2060	0.9915	0.030	0.100	−11.91	5.2	5.2
	AFM1	0.020–50	y = 4895x − 1050	0.9992	0.006	0.020	−18.99	3.0	5.9

### 3.9 Method validation

The analytical performance of the method was validated in accordance with Regulation (EU) 2023/2782 through the assessment of several key parameters, including its linearity, determination coefficients (*R*^*2*^), limits of detection (LODs), limits of quantitation (LOQs), and precision (repeatability and reproducibility). Instrument performance and measurement uncertainty were also assessed. The LODs and LOQs were calculated at a signal-to-noise ratio (S/N) of 3 and 10, respectively. To eliminate the potential MEs, matrix-matched calibration curves were employed for quantification across the nine food matrices. Measurement uncertainty was estimated from method validation data. The standard uncertainty (*u*) was calculated as the following formula (Eq. (3)):

(3) $$u={\left[{\left(\mathrm{LOD}/2\right)}^{2}+{\left(\mathrm{\alpha C}\right)}^{2}\right]}^{1/2}$$

where LOD is the method's limit of detection for the specific analyte and matrix; *C* is the analyte concentration; and *α* is the within-laboratory reproducibility relative standard deviation (RSD). The expanded uncertainty (*U*) was derived using a coverage factor of *k* = 2, (95 % confidence). The final results are recorded as *C* ± *U*.

As shown in Table 1, good linearity (*R*^*2*^ ≥ 0.9892) was achieved for all analytes. LODs ranged from 0.001 to 0.045 μg kg^−1^. Notably, all LOQs (0.010–0.150 μg kg^−1^) met the stricter guideline of the LOQ not exceeding 0.2 times the maximum residue limits (MRLs), as required by EU Regulation 2023/2782. A statistical analysis further revealed that method sensitivity was analyte-dependent (ANOVA, *F* = 7.253, *p* < 0.001; Table S4), with AFB1 and AFM1 having lower LODs than the G-group toxins. This performance variation can be attributed to inherent structural differences influencing key analytical parameters such as the MSPE efficiencies and the instrumental response.

The precision of the method was validated by comparing the experimental repeatability (intra-day precision, RSD_*r*_) and reproducibility (inter-day precision, RSD_*R*_) against the predicted thresholds derived from the Horwitz equations (described in Supplementary Material) (Baltacı, İlyasoğlu, & Yüksel, 2013). For the 5 μg kg^−1^ spiking level, the Horwitz equation predicts acceptable limits of ≤23.5 % for repeatability (PRSD_*r*_) and ≤35.6 % for reproducibility (PRSD_*R*_). As shown in Table 1, the experimental RSD_*r*_ (≤9.4 %) and RSD_*R*_ (≤10.5 %) were less than half of the predicted thresholds, confirming the excellent precision and reliability of the analytical procedure.

To further access the accuracy of the method, the spiked recovery experiments were conducted. As presented in Table S5, the recoveries varied from 71.5 % and 112.8 % with RSD values were less than 5.6 %. Furthermore, the experimental recoveries were evaluated against performance criteria from EU guidelines at three spiking levels. At 0.5 μg kg^−1^, recoveries for B/G-aflatoxins (71.5 %–112.8 %) and AFM1 (72.2 %–105.2 %) were fully compliant with their respective 50 %–120 % and 70 %–110 % acceptance ranges. At 5.0 μg kg^−1^, the recoveries (73.8 %–109.6 %) met the required 70 %–110 % for all analytes. At 30 μg kg^−1^, the majority of recoveries met the established 80–110 % criterion. Minor deviations were found for AFG1 in oat (77.1 %) and for AFG2 in banana (73.8 %), which were marginally below the lower limit. Overall, the good recovery results across a wide range of matrices and concentrations, despite these few slight deviations, demonstrated the high accuracy and reliability of the proposed method.

To ensure the reliability of the HPLC-MS/MS instrument, a system suitability test was also performed by injecting a quality control standard solution (5 μg kg^−1^) at the beginning of each analytical sequence and after every 10 samples. The stability of retention times (RSD < 0.9 %) and peak area responses (RSD < 2.5 %) confirmed the instrument's consistent and robust performance throughout the analyses.

### 3.10 Actual sample analysis

The practical applicability of the method was evaluated by analyzing AFs from cereals and cereal product (corn, rice, soybean, sweet potato, oats and bread), fruits (banana), edible oil (colleseed oil) and milk (whole fat liquid milk) samples. The measured concentration was 0.9 μg kg^−1^. In accordance with Regulation (EU) 2023/2782, this result was corrected for a mean recovery of 76.1 % (determined at a 0.5 μg kg^−1^ spiking level), the final corrected concentration is 1.18 ± 0.19 μg kg^−1^ (expanded uncertainty, *k* = 2). This level is not exceeding the maximum residue limits set for corn by both China (20 μg kg^−1^, GB 2761-2017, 2017) and the EU (2 μg kg^−1^; Regulation (EU) 2023/2782), respectively. No AFs were detected in the other analyzed samples.

### 3.11 Method comparison

The developed 4F-COF@Fe_3_O_4_-based MSPE method was compared with previously reported methods (Table S6), focusing on critical parameters such as adsorption time, desorption time, adsorbent dosage, eluent solvent consumption, LODs, and recoveries. The synthesis of 4F-COF@Fe_3_O_4_ utilizes cost-effective, readily accessible building units via a simple one-pot in-situ strategy, enhancing its practical applications. Compared to other sample preparation techniques (Wei et al., 2025; Wei et al., 2023; Wang et al., 2024; Fang et al., 2025; Wang et al., 2023; Xu, Sun, Wang, Zhang, & Sun, 2021; Li, Lv, et al., 2022; Li, Xu, et al., 2022), the newly developed method requires a small sample volume (1 mL), a minimal adsorbent dosage (2 mg) with rapid adsorption (15 s) and desorption (3 min). Furthermore, its LODs (0.001–0.16 μg kg^−1^) and recoveries (71.5 %–112.8 %) are comparable or superior to other reported methods. The experimental and comparative findings confirm the high sensitivity and accuracy of the proposed method.

## 4 Conclusions

In summary, this study aimed to address the limitations in determination of trace AFs in diverse food matrices. To this end, a fluorine-rich magnetic COF (4F-COF@Fe_3_O_4_) was rationally designed and conveniently prepared via a one-pot approach. This material exhibited desirable properties, including high surface area and porosity, strong magnetic responsiveness, and remarkable stability and reusability. Experimental and DFT investigations revealed that the superior extraction efficiency towards AFs was primarily attributed to the fluorine-mediated interaction mechanisms, such as fluorine-driven van der Waals forces, π–π stacking, hydrogen bonding, electrostatic interactions, and pore-matching effects. The developed 4F-COF@Fe_3_O_4_-MSPE-HPLC-MS/MS method demonstrated satisfactory sensitivity, linearity, and reproducibility for trace AFs detection across diverse food matrices, offering rapid extraction with minimal solvent and adsorbent consumption. These findings highlight the potential of 4F-COF@Fe_3_O_4_ as a superior and valuable adsorbent for enhancing trace AFs monitoring in wide range of food samples, significantly contributing to food safety control.

## CRediT authorship contribution statement

**Dan Wei:** Writing – original draft, Validation, Resources, Project administration, Methodology, Funding acquisition, Data curation, Conceptualization. **Jianliang Li:** Writing – original draft, Software, Resources, Formal analysis. **Yixuan Ni:** Validation, Methodology. **Qiao Deng:** Validation, Resources, Formal analysis. **Ming Guo:** Validation, Methodology, Data curation. **Zuxin Wang:** Writing – review & editing, Software. **Huizhen Wu:** Visualization, Resources, Methodology, Funding acquisition. **Xu Wang:** Visualization, Supervision, Funding acquisition, Formal analysis. **Jingjing Xu:** Validation, Resources, Methodology, Formal analysis, Conceptualization.

## Declaration of competing interest

The authors declare that they have no known competing financial interests or personal relationships that could have appeared to influence the work reported in this paper.

## Data Availability

Data will be made available on request.
