# Supplementary material for: A highly fluorinated functionalized magnetic covalent organic framework for high-efficient extraction of aflatoxins from diverse food matrices
Source: Food Chem X. 2025 Aug 5;30:102863. doi: 10.1016/j.fochx.2025.102863 (PMC12396029; doi:10.1016/j.fochx.2025.102863)
Supplement: Supplementary material [file mmc1.docx]

**A highly fluorinated functionalized magnetic covalent organic framework for high-efficient extraction of aflatoxins from diverse food matrices**

Dan Wei^1, 2^, Jianliang Li^1^, Yixuan Ni^1^, Qiao Deng^1^, Ming Guo^4^, Zuxin Wang^5^, Huizhen Wu^3*^, Xu Wang^1, 2*^, Jingjing Xu^1*^

*^1^School of Laboratory Medicine and Bioengineering, Hangzhou Medical College, Hangzhou, 310053, China, Zhejiang, 311300, China*

*^2^Key Laboratory of Biomarkers and In Vitro Diagnosis Translation of Zhejiang province, Hangzhou, 310058, China*

*^3^College of Biology and Environmental Engineering, Zhejiang Shuren University, Hangzhou, 310015, China*

*^4^Zhejiang Chemical Production Quality Inspection Co., Ltd, Hangzhou, 310023, China*

*^5^School of Economics and Management, Universiti Putra Malaysia, Kampong Serdang Belah Selangor, 43000, Malaysia*

*Corresponding authors:

1. *mail address:* [jingjingxu@hmc.edu.cn](mailto:wangxu@hmc.edu.cn) (Jingjing Xu) & wuhuizhen@zjsru.edu.cn (Huizhen Wu) & [wangxu@hmc.edu.cn](mailto:wangxu@hmc.edu.cn) (Xu Wang)

Contents

[1. Equations S3](#_Toc10446)

[1.1. Langmuir model: S3](#_Toc27566)

[1.2. Freundlich model: S3](#_Toc9723)

[1.3. Quasi-first-order kinetic model: S3](#_Toc26494)

[1.4. Quasi-second-order kinetic model S3](#_Toc9765)

[1.5. Horwitz equation S3](#_Toc9765)

[2. Supporting tables S4](#_Toc9038)

[Table S1 S4](#_Toc30827)

[Table S2 S4](#_Toc27513)

[Table S3 S4](#_Toc29323)

[Table S4 S5](#_Toc22101)

[Table S5 S6](#_Toc13030)

[Table S6 S8](#_Toc13030)

[3. Supporting figures S9](#_Toc27795)

[Fig. S1. S9](#_Toc23519)

[Fig. S2. S10](#_Toc22969)

[Fig. S3. S11](#_Toc23261)

[Fig. S4. S12](#_Toc23261)

[Fig. S5. S13](#_Toc3462)

[Fig. S6. S14](#_Toc8381)

[Fig. S7. S15](#_Toc15665)

## 1. Equations

## 1.1. Langmuir model:

 (1)

where *K_L_* (L mg) is the constants of the Langmuir. *C_e_* (mg L^-1^) is the concentration at equilibrium, *Q_e_* (mg g^-1^) is adsorption capacity at equilibrium and *Q_m_* (mg g^-1^) is the maximum adsorption capacity.

## 1.2. Freundlich model:

 (2)

where *K_F_* (mg g (mg L)^-1/n^) are the constants of the Freundlich model, *Q_e_* (mg g^-1^) and *C_e_* (mg L^-1^) is the adsorption capacity and concentration at equilibrium.

## 1.3. Quasi-first-order kinetic model:

 (3)

## 1.4. Quasi-second-order kinetic model

 (4)

where *Q_e_* and *Q_t_* are the adsorption capacities at equilibrium and at t time, respectively, and *K_1_* and *K_2_* are the rate constants for the quasi-first-order reaction and quasi-second-order reaction models, respectively.

## 1.5. Horwitz equation

PRSD*_R_* = 2 ^(1 - 0.5 log^ *^C^*^)^  (5)

PRSD*_r_ =* 0.66 × PRSD*_R_* (6)

where *C* is the concentration ratio (i.e. 1 = 100g/100g, 0.001 = 1000 mg/kg).

## 2. Supporting tables

**Table S1**

MS parameters for detection of AFs.

| Compound | Precursor ion (m/z) | Product ion (m/z) | Fragmentor  (V) | Collision energy (eV) | Polarity |
| --- | --- | --- | --- | --- | --- |
| AFB1 | 313 | 285 | 160 | 20 | Positive |
| AFB2 | 315 | 287 | 160 | 25 | Positive |
| AFG1 | 329 | 243 | 160 | 25 | Positive |
| AFG2 | 331 | 313 | 160 | 25 | Positive |
| AFM1 | 329 | 273 | 166 | 30 | Positive |

**Table S2**

The adsorption parameters for the Langmuir model and the Freundlich model

| Langmuir isotherm | | | Freundlich isotherm | | |
| --- | --- | --- | --- | --- | --- |
| *Q_m_* | *K_L_* | *R^2^* | *1/n* | *K_F_* | *R^2^* |
| 185.16 | 11.43 | 0.9836 | 0.79 | 0.43 | 0.9744 |

**Table S3**

The adsorption parameters for the pesudo-first-order and the pesudo-second-order kinetic models

| Pseudo first-order | | | Pseudo second-order | | |
| --- | --- | --- | --- | --- | --- |
| *Q_e_* | *K_1_* | *R^2^* | *Q_e_* | *K^2^* | *R^2^* |
| 0.0005 | 0.72 | 0.3198 | 2.26 | 4.60 | 0.9694 |

**Table S4**

Tukey HSD post-hoc analysis for pairwise comparison of mean LODs (µg kg^-1^) among five aflatoxins across nine matrices.

| Analyte 1 | Analyte 2 | Mean Difference (1-2) | Standard Error | *p*-value | 95% Confidence Interval | |
| --- | --- | --- | --- | --- | --- | --- |
|  |  |  |  |  | Lower | Higher |
| AFB1 | AFB2 | -0.00978 | 0.00355 | 0.064 | -0.0199 | 0.0004 |
|  | AFG1 | -.01633* | 0.00355 | < 0.001 | -0.0265 | -0.0062 |
|  | AFG2 | -.01400* | 0.00355 | 0.003 | -0.0241 | -0.0039 |
|  | AFM1 | -0.00422 | 0.00355 | 0.758 | -0.0144 | 0.0059 |
| AFB2 | AFB1 | 0.00978 | 0.00355 | 0.064 | -0.0004 | 0.0199 |
|  | AFG1 | -0.00656 | 0.00355 | 0.363 | -0.0167 | 0.0036 |
|  | AFG2 | -0.00422 | 0.00355 | 0.758 | -0.0144 | 0.0059 |
|  | AFM1 | 0.00556 | 0.00355 | 0.529 | -0.0046 | 0.0157 |
| AFG1 | AFB1 | .01633* | 0.00355 | < 0.001 | 0.0062 | 0.0265 |
|  | AFB2 | 0.00656 | 0.00355 | 0.363 | -0.0036 | 0.0167 |
|  | AFG2 | 0.00233 | 0.00355 | 0.964 | -0.0078 | 0.0125 |
|  | AFM1 | .01211* | 0.00355 | 0.012 | 0.002 | 0.0223 |
| AFG2 | AFB1 | .01400* | 0.00355 | 0.003 | 0.0039 | 0.0241 |
|  | AFB2 | 0.00422 | 0.00355 | 0.758 | -0.0059 | 0.0144 |
|  | AFG1 | -0.00233 | 0.00355 | 0.964 | -0.0125 | 0.0078 |
|  | AFM1 | 0.00978 | 0.00355 | 0.064 | -0.0004 | 0.0199 |
| AFM1 | AFB1 | 0.00422 | 0.00355 | 0.758 | -0.0059 | 0.0144 |
|  | AFB2 | -0.00556 | 0.00355 | 0.529 | -0.0157 | 0.0046 |
|  | AFG1 | -.01211* | 0.00355 | 0.012 | -0.0223 | -0.002 |
|  | AFG2 | -0.00978 | 0.00355 | 0.064 | -0.0199 | 0.0004 |

The mean difference is significant at the *p* < 0.05 level

**Table S5**

Recoveries of aflatoxins in real samples (n =3)

| Analytes | Spiked  (μg kg^-1^) | Corn | | Rice | | Soybean | | Sweet potato | | Oats | | Banana | | Colleseed oil | | Milk | | Bread | |
| --- | --- | --- | --- | --- | --- | --- | --- | --- | --- | --- | --- | --- | --- | --- | --- | --- | --- | --- | --- |
|  |  | Recovery  (%) | RSD  (%) | Recovery  (%) | RSD  (%) | Recovery  (%) | RSD  (%) | Recovery  (%) | RSD  (%) | Recovery  (%) | RSD  (%) | Recovery  (%) | RSD  (%) | Recovery  (%) | RSD  (%) | Recovery  (%) | RSD  (%) | Recovery  (%) | RSD  (%) |
| AFB1 | 0.5  5.0  30 | 76.1±2.1  82.8±0.7  83.5±0.6 | 1.3  3.2  2.5 | 89.2±1.3  101.1±1.0  93.1±2.7 | 3.1  0.9  1.1 | 85.6±2.8  79.9±3.3  90.0±4.5 | 2.6  1.9  1.5 | 87.9±1.8  93.2±1.6  98.6±1.6 | 2.5  3.1  0.8 | 106.3±3.5  94.6±2.1  94.9±3.5 | 1.2  2.1  3.7 | 90.1±3.1  89.6±1.7  91.2±3.0 | 2.3  1.6  2.8 | 93.1±2.3  90.8±0.7  92.5±1.1 | 2.3  1.1  3.5 | 81.1±0.9  79.2±2.6  90.5±2.0 | 0.9  1.2  1.9 | 80.9±2.1  90.2±3.1  101.0±4.5 | 2.3  3.8  1.0 |
| AFB2 | 0.5  5.0  30 | 80.5±1.9  85.7±2.2  90.6±2.1 | 3.6  5.6  3.6 | 79.2±3.6  90.0±4.1  88.9±1.5 | 3.2  2.0  1.6 | 83.2±2.8  90.2±0.7  89.9±2.0 | 1.6  0.8  2.1 | 77.1±2.5  90.6±0.7  89.7±3.3 | 1.5  3.7  2.1 | 73.2±3.0  80.5±2.6  90.3±1.1 | 2.5  1.8  2.0 | 112.8±2.3  109.6±4.1  92.1±2.0 | 0.8  1.9  1.5 | 85.2±3.1  98.1±2.6  100.2±3.0 | 0.6  1.9  1.6 | 90.5±1.6  89.6±3.7  93.1±2.6 | 3.6  2.5  4.7 | 90.5±2.9  85.9±3.6  82.1±3.2 | 3.0  3.1  1.6 |
| AFG1 | 0.5  5.0  30 | 78.9±3.6  86.1±1.7  90.1±3.5 | 2.7  2.1  0.8 | 78.5±3.9  88.9±3.1  90.1±3.9 | 1.5  0.7  2.1 | 88.1±1.5  89.6±2.5  93.5±3.0 | 4.2  2.1  1.9 | 83.1±1.3  92.5±4.5  89.9±2.3 | 3.6  4.2  1.9 | 76.4±4.1  73.8±3.7  77.1±2.1 | 1.0  0.9  1.5 | 90.3±2.1  87.1±3.8  90.5±1.7 | 1.9  2.7  2.8 | 74.7±2.2  78.5±5.0  80.0±3.6 | 2.5  3.6  1.6 | 92.1±3.1  78.6±1.7  83.1±5.1 | 1.1  0.8  2.7 | 95.8±4.6  101.2±3.3  109.1±1.2 | 5.2  2.9  3.1 |
| AFG2 | 0.5  5.0  30 | 71.5±2.0  89.0±3.1  81.5±3.8 | 3.0  1.3  2.1 | 83.5±2.8  82.1±3.5  90.1±2.2 | 0.9  3.6  1.8 | 90.8±1.6  103.2±0.7  93.5±1.9 | 1.7  2.6  3.1 | 78.2±2.0  79.7±0.7  83.1±1.7 | 3.8  3.7  1.6 | 85.7±2.5  83.1±1.6  90.0±3.8 | 3.2  1.3  0.9 | 75.1±2.5  73.9±3.1  73.8±3.5 | 1.6  3.1  2.3 | 83.3±3.1  90.1±0.8  89.9±1.8 | 2.0  1.9  3.1 | 77.2±2.2  80.1±1.0  86.8±5.3 | 2.2  0.9  2.5 | 79.9±2.8  80.5±2.1  90.6±3.5 | 3.9  2.1  2.5 |
| AFM1 | 0.5  5.0  30 | 72.2±1.9  76.2±2.1  82.3±0.9 | 2.2  1.9  3.6 | 105.2±3.8  93.1±3.8  92.1±2.1 | 2.9  2.1  1.9 | 91.5±3.8  93.1±2.9  95.0±2.5 | 0.8  1.2  6.8 | 90.6±2.5  102.1±5.1  89.8±5.9 | 2.1  1.0  3.1 | 90.5±0.8  88.8±2.6  92.2±2.8 | 2.1  3.1  1.0 | 83.0±2.8  85.1±1.9  90.0±2.9 | 3.6  0.8  1.1 | 100.5±4.2  96.1±2.5  93.5±1.9 | 1.0  1.2  2.1 | 85.2±2.7  93.8±1.9  103.1±1.7 | 1.6  0.9  2.1 | 89.5±5.2  95.8±0.9  91.6±2.1 | 2.1  3.2  2.9 |

**Table S6**

Compare with other methods for the detection of AFs

| Sample | Analyte | Method | Adsorbent | Adsorbent dosage  (mg) | Volume  of eluent  (mL) | Extraction time (s) | Linearity (µg kg^-1^) | Recovery  (%) | Reference |
| --- | --- | --- | --- | --- | --- | --- | --- | --- | --- |
| Maize | AFB1, AFB2, AFG1, AFG2 | MSPE/HPLC-MS/MS | Fe_3_O_4_/COF-TpBD | 5 | 1 | 30 | 0.5-50 | 75.5-96.8 | (Wei et al., 2023) |
| Rice, oatmeal, milk | AFB1, AFB2, AFG1,AFG2, AFM1 | MSPE/HPLC-MS/MS | Fe_3_O_4_@TAPT-OH-COFs | 5 | 1 | 240 | 0.05-20 | 75.3-110.9 | (Wei, et al., 2025) |
| Nuts | AFB1, AFB2, AFG1, AFG2 | SPE/HPLC-FLD | F-COF | 35 | 0.2 | 2040 | 0.08-16.0 | 83.5-114.0 | (Wang et al., 2024) |
| Peanuts | AFB1, AFB2, AFG1, AFG2 | SPE/HPLC-FLD | TF-COF | 30 | 0.3 | >1500 | 0.025-20 | 86.3-116 | (Fang, et al., 2025) |
| Fruit (tomato) | AFB1 | MSPE/UHPLC–MS/MS | Fe_3_O_4_@COF(TAPT–DHTA) | 20 | 3 | 480 | 0.5-200 | 77.2-83.6 | (Wang et al., 2023) |
| Vegetable oils | AFB1, AFB2, AFG1, AFG2 | MSPE/HPLC/FLD | PDA@Fe_3_O_4_-MWCNTs | 50 | 2 | 600 | 1-50 | 70.2-89.3 | (Xu et al., 2021) |
| Milk, edible oil, and rice | AFB1, AFB2, AFG1, AFG2 | MSPE/HPLC–MS/MS | Fe_3_O_4_@COF  (TFPB-PPD) | 2 | - | >300 | 0.1-20 | 76-83 | (Li, et al. 2022) |
| 9 food matrices | AFB1, AFB2, AFG1, AFG2,  AFM1 | MSPE/HPLC-MS/MS | 4F-COF@Fe_3_O_4_ | 2 | 1 | 15 | 0.01-50 | 71.5-112.8 | This work |

MSPE: magnetic-solid phase extraction; BIDDT: binding-induced DNA dissociation technique; FLD: fluorescence detection; SPE: solid phase extraction; UPLC: ultra performance liquid chromatography; PCD: photochemical derivatization

## Supporting figures


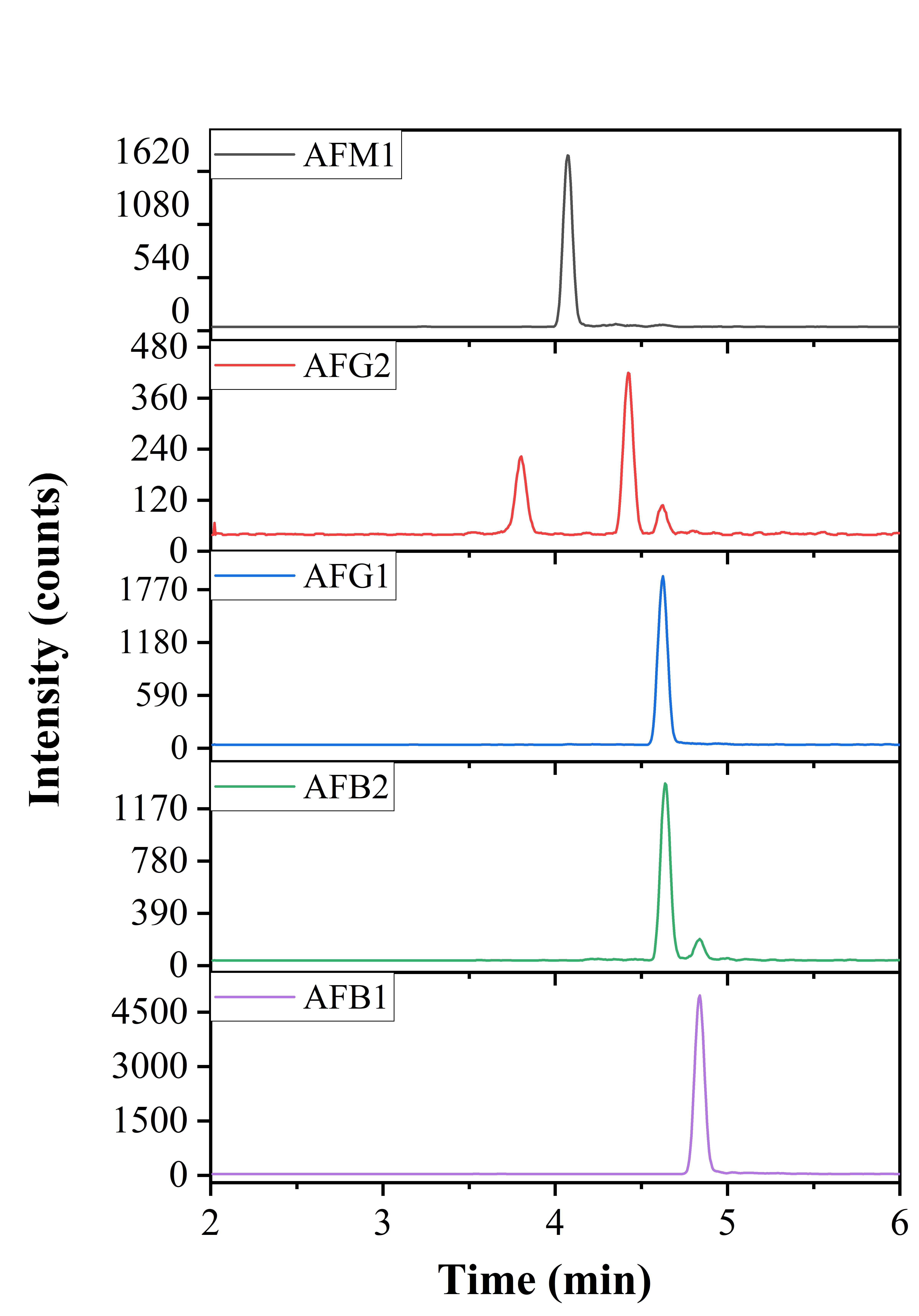


Fig. S1. MRM chromatogram of the mixed AFs standard at a concentration of 5 µg kg^-1^.


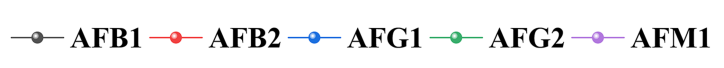





Fig. S2. The reusability of the 4F-COF@Fe_3_O_4_


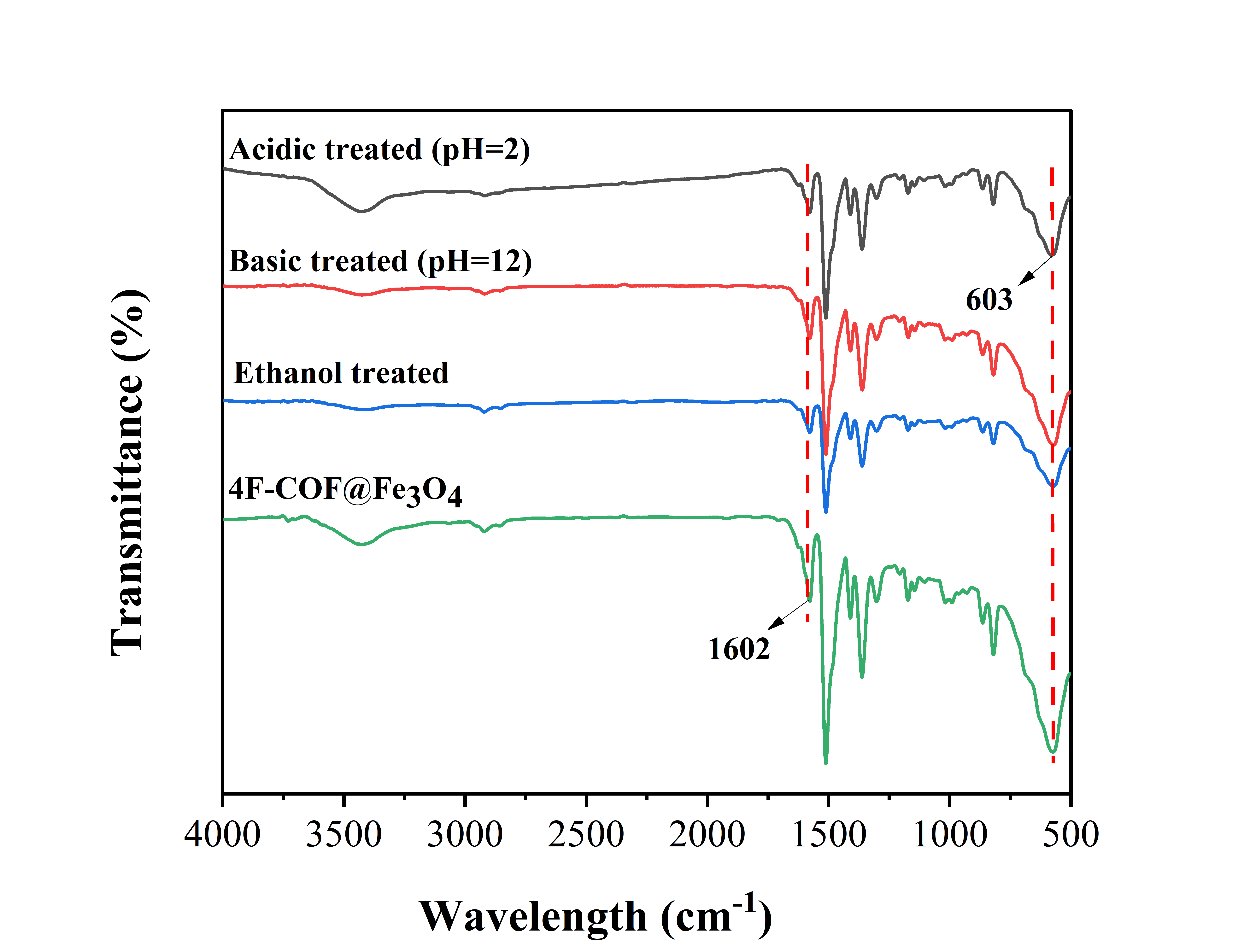


Fig. S3. FTIR spectra of 4F-COF@Fe_3_O_4_ after acid, base and organic solvent treatment.

**
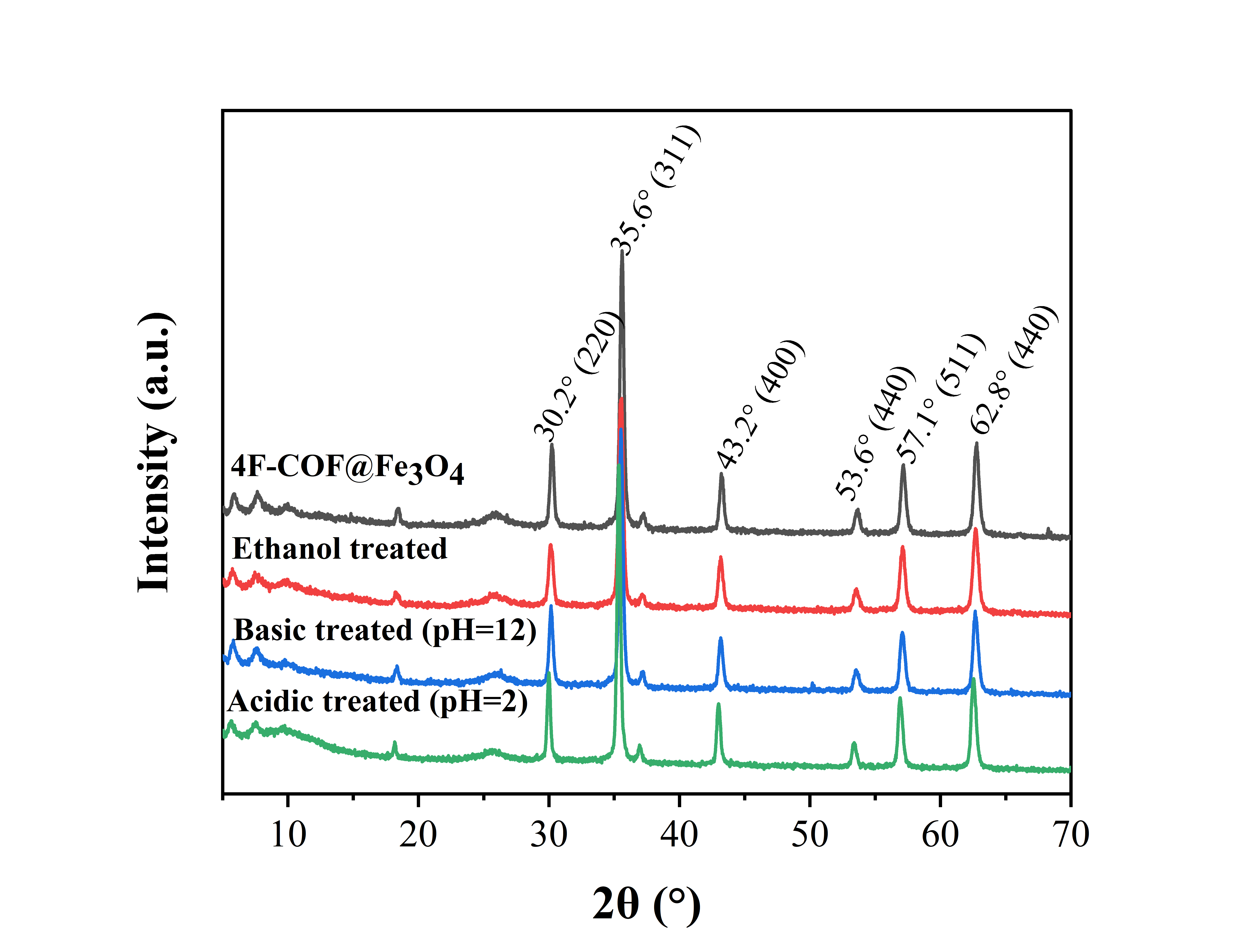
**

a

**
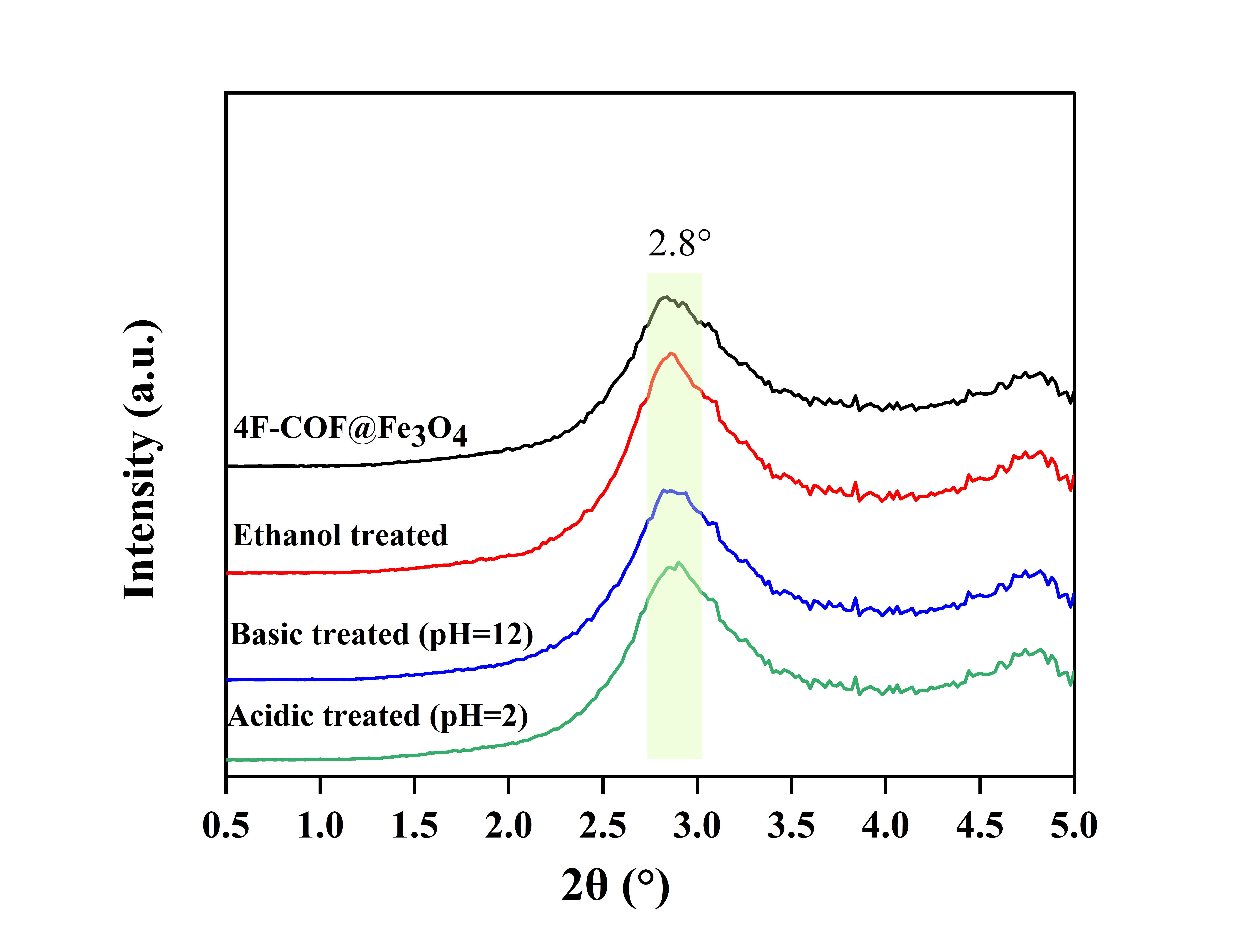
**

b

Fig. S4. Wide-Angle (a) and low-Angle (b) XRD patterns of 4F-COF@Fe_3_O_4_ after acid, base and organic solvent treatment.

**
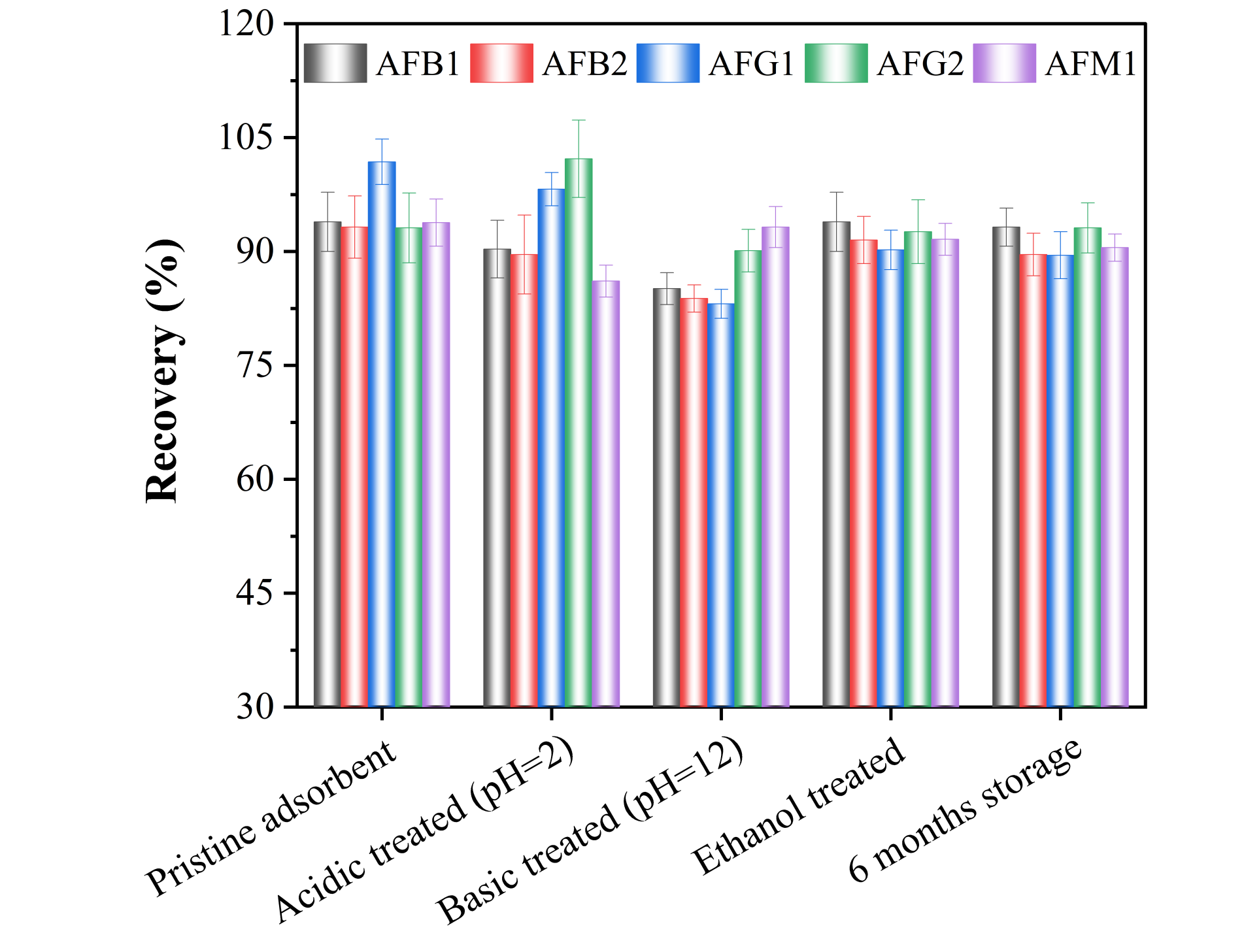
**

Fig. S5. The reproducibility of 4F-COF@Fe_3_O_4._


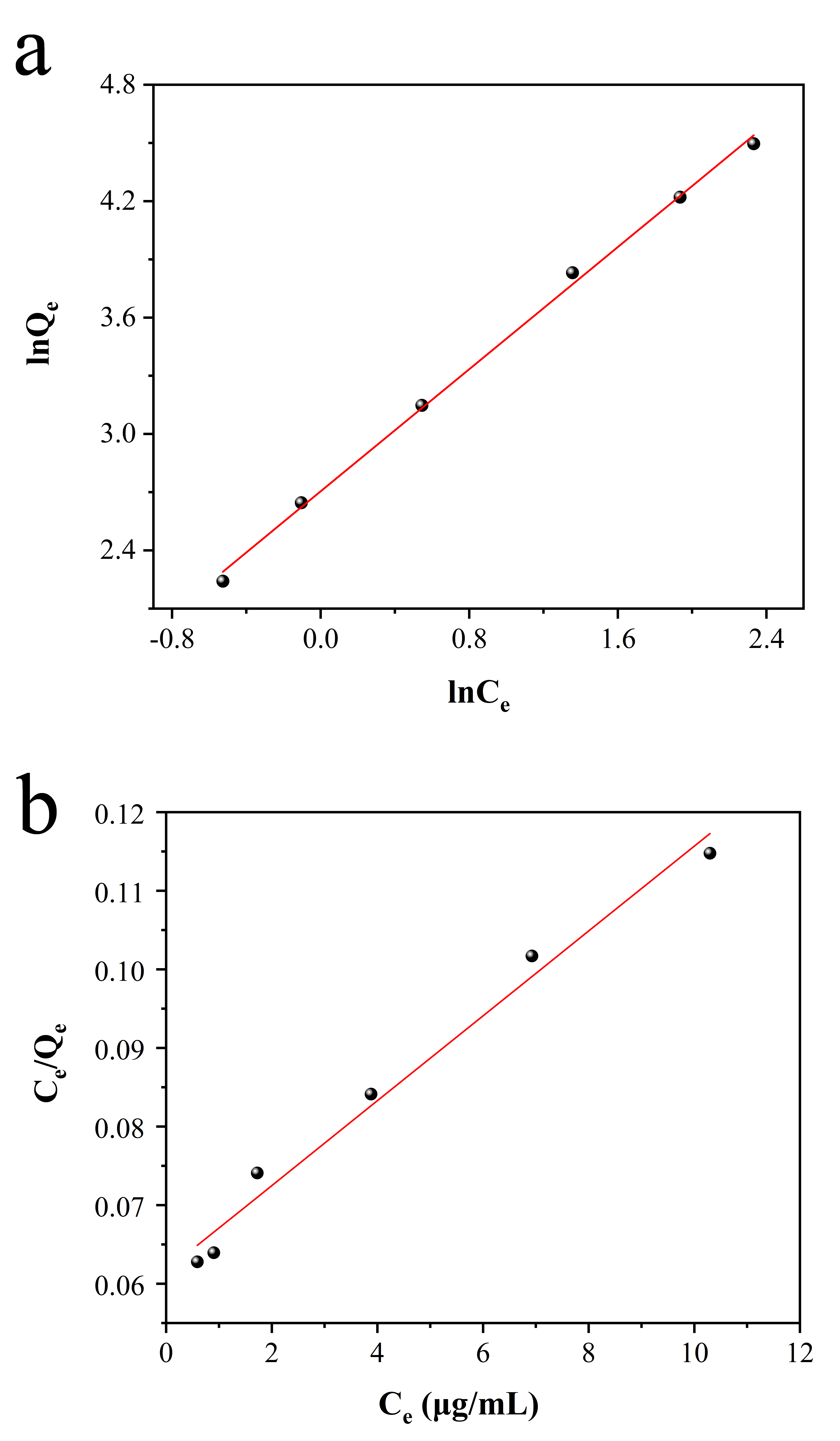


Fig. S6. (a) The Freundlich model and (b) the Langmuir model.


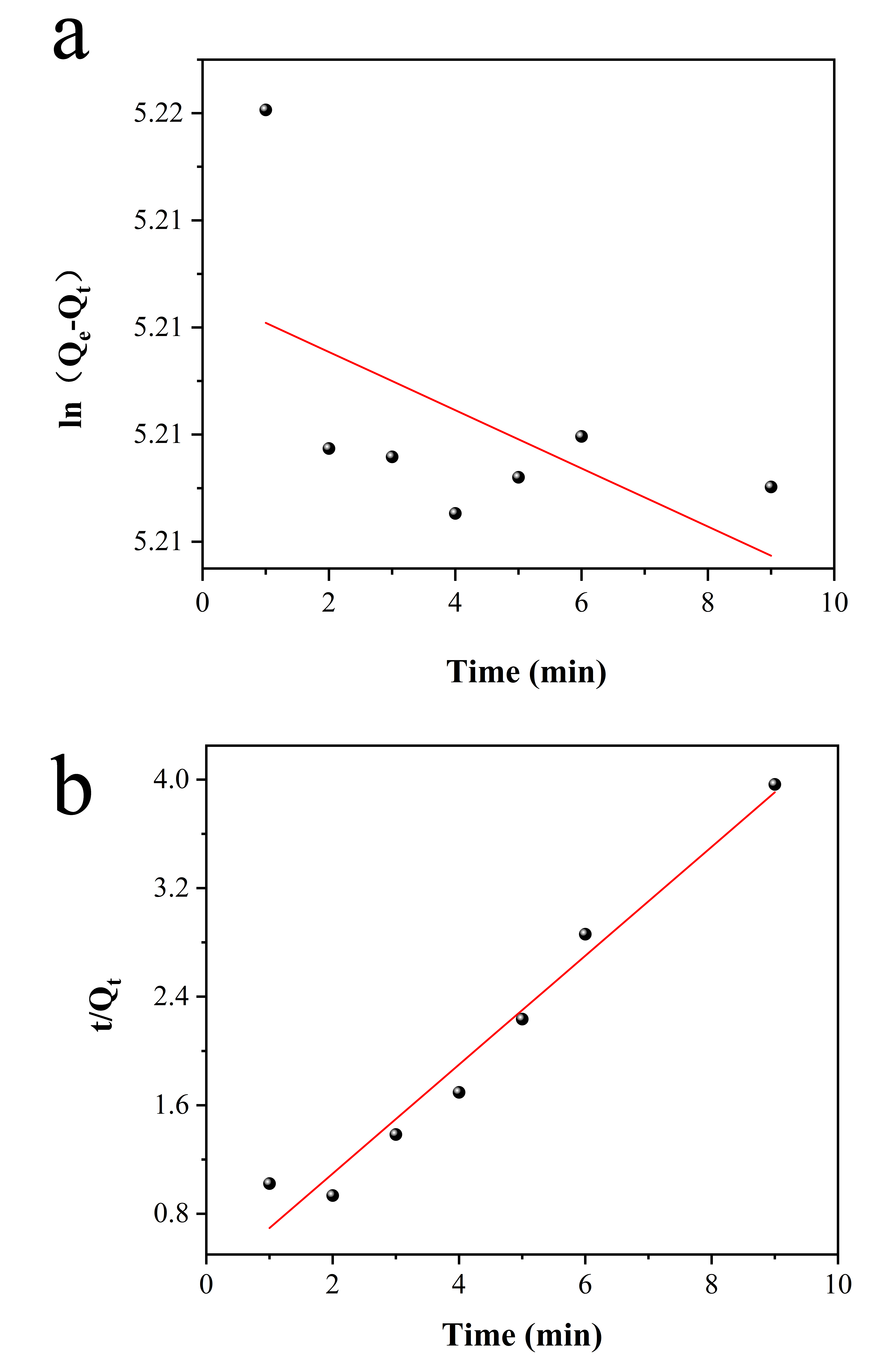


Fig. S7. (a) The pseudo-first-order kinetic model fitting curve and (b) the pseudo second order kinetic model fitting curve.
